# Supplementary material for: A Bayesian Hierarchical Mixture Cure Modelling Framework to Utilize Multiple Survival Datasets for Long‐Term Survivorship Estimates: A Case Study From Previously Untreated Metastatic Melanoma
Source: Stat Med. 2025 May 30;44(13-14):e70132. doi: 10.1002/sim.70132 (PMC12124107; doi:10.1002/sim.70132)
Supplement: Supplementary file 1 — Data S1. Supporting Information. [file SIM-44-0-s001.pdf]

Received: Added at production | Revised: Added at production | Accepted: Added at production

DOI: xxx/xxxx

ARTICLE TYPE

APPENDIX: A Bayesian hierarchical mixture cure modelling framework to utilize multiple survival datasets for long-term survivorship estimates: A case study from previously untreated metastatic melanoma

Nathan Green*[1] | Murat Kurt[2] | Andriy Moshyk[2] | James Larkin[3] | Gianluca Baio[1]

[1]Department of Statistical Science, UCL, London, UK
[2]Worldwide Health Economics & Outcomes Research, Bristol Myers Squibb, NJ, USA
[3]The Royal Marsden Hospital, London, UK

Correspondence
*Nathan Green Email: n.green@ucl.ac.uk

1 | MODEL FORMULA

The common hierarchical cure fraction model likelihood for treatment k without background risk is

$$L(\pi, \theta) = \prod_{i=1}^{n} \prod_{j=1}^{J} \left[(1 - \pi_{kj}) f_u(t_{ij} \mid \theta_j^u, x_i) \right]^{\delta_{ij}} \left[\pi_{kj} + (1 - \pi_{kj}) S_u(t_{ij} \mid \theta_j^u, x_i) \right]^{1-\delta_{ij}} \cdot g(\pi_{kj} \mid \pi_k)$$

where

- π is the cure fraction
- $f_u()$ is the pdf for uncured patients
- $S_u()$ is the survival function for uncured patients
- δ_i is the censoring indicator
- $g()$ is the hierarchical density for the cure fraction

To include the background risk, f_u is replaced with the combined pdf $(h_u + h_b)S_u S_b$ and the censored time likelihood component is multiplied by S_b to give

$$L(\pi, \theta) = \prod_{i=1}^{n} \prod_{j=1}^{J} \left[(1 - \pi_{kj}) \left(h_u(t_{ij} \mid \theta_j^u, x_i) + h_b(t_{ij} \mid \theta^b, x_i) \right) S_u(t_{ij} \mid \theta_j^u, x_i) S_b(t_{ij} \mid \theta^b, x_i) \right]^{\delta_{ij}}$$
$$\times \left[S_b(t_{ij} \mid \theta^b, x_i) \left(\pi_{kj} + (1 - \pi_{kj}) S_u(t_{ij} \mid \theta_j^u, x_i) \right) \right]^{1-\delta_{ij}} \times g(\pi_{kj} \mid \pi_k). \quad (1)$$

2 | USE OF WORLD HEALTH ORGANISATION (WHO) LIFE TABLES

We used the World Health Organisation (WHO) life tables by country for the latest year available of 2016[?] to inform the background mortality in the mixture cure model. The mortality data provide a comprehensive look-up table of hazards by age, sex and country. We can consider this as a 3-dimensional array h^{WHO}. The following diagram illustrates this structure.

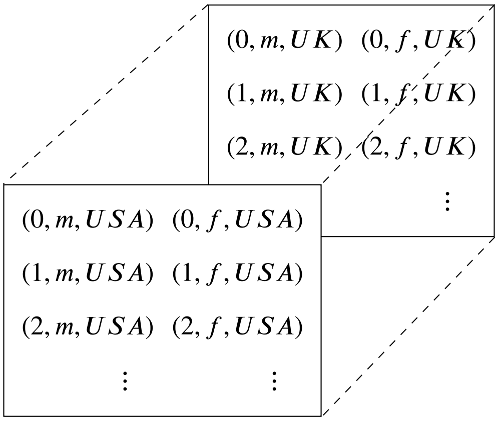

The baseline hazards are the expected mortality rate for each patient at the age at which they experience the event, e.g. $h_j(i) = h^{WHO}(age_j(i), sex(i), country(i))$, for individual i and end-point j.

Figure 1 shows the WHO life table survival curves for each country and sex.

3 | CURE FRACTION PRIOR DISTRIBUTIONS

Figures 2 and 3 show prior predictive distributions for the cure fractions. Figure 3 assumes an exponential distribution with rate 1 for the uncured fraction and survival probability 1 for the cured fraction. This is purely for demonstration purposes since the behaviour we wish to show is how the Kaplan-Meier curves change depending on the prior sampled cure fractions.

The same prior distribution is used for all of the treatment cure fractions. Further structure could be imposed in the model. We would specify a prior with larger mean value for the combined treatment or further force the ordering of cure fractions such that the combined treatment cure fraction is necessarily larger than the monotherapy cure fractions.

We found that this was unnecessary for this analysis however. From Figures 2 and 3 the end-point-specific prior distributions are fairly vague and, as can be seen in the main analysis results, the posterior distributions were consistent with such assumptions.

4 | LATENT MODEL PRIOR DISTRIBUTIONS

Figures 4 shows Kaplan-Meier curves using realisations from the latent model parameter prior distribution and an exponential survival distribution. We assume everyone has the median age of 58 years for simplicity. We can see that the spread of survival curves is quite wide with median event times from about 5 months to about 35 months. To clarify this change in the distribution of survival probabilities at specific time points, Figure 5 shows histograms at 30, 40, 50 and 60 months.

5 | POSTERIOR MEAN SURVIVAL CURVES

6 | POSTERIOR PARAMETER STATISTICS

7 | FOREST PLOTS

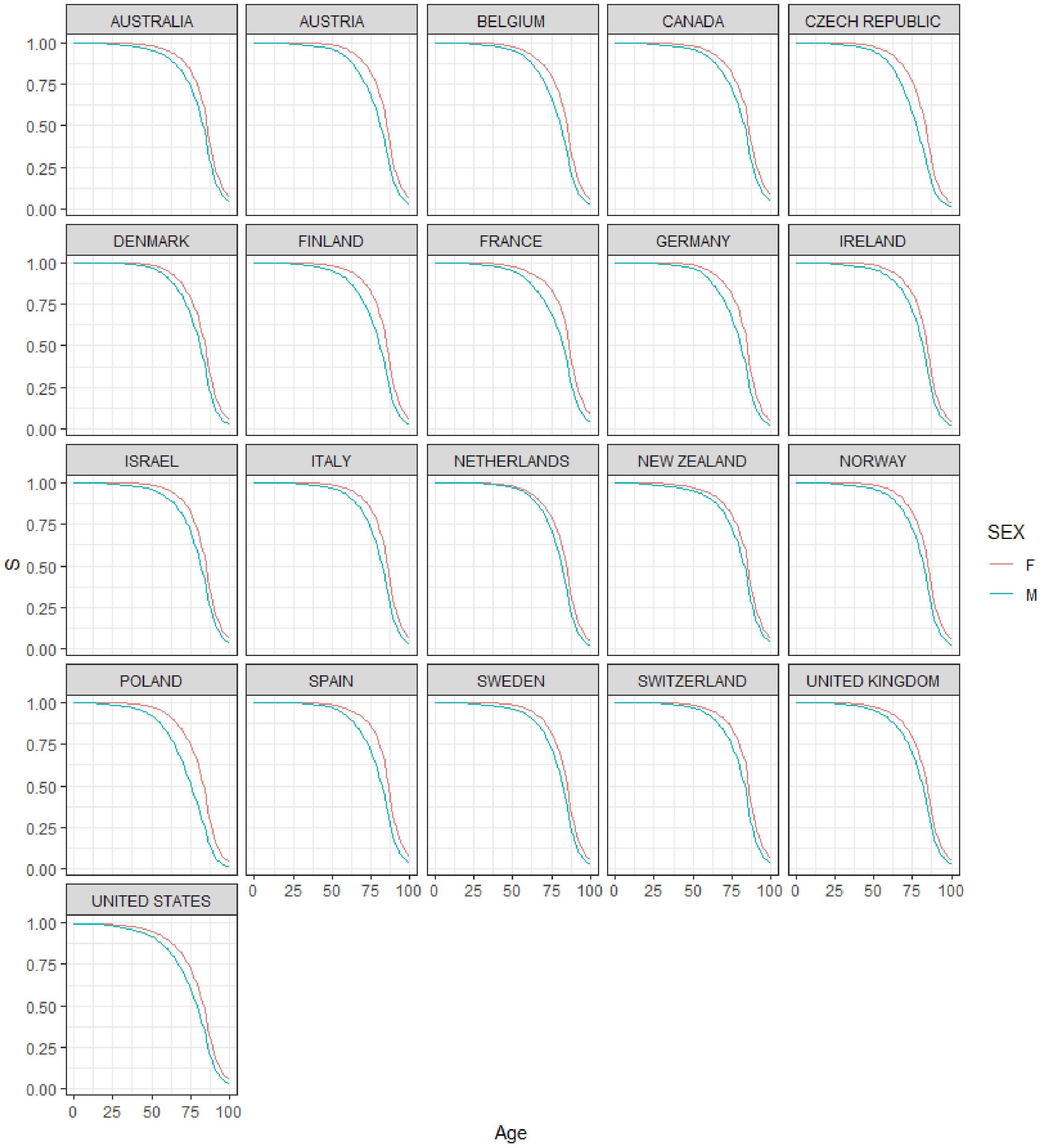

Figure 1 WHO life table survival curves for each country and sex.

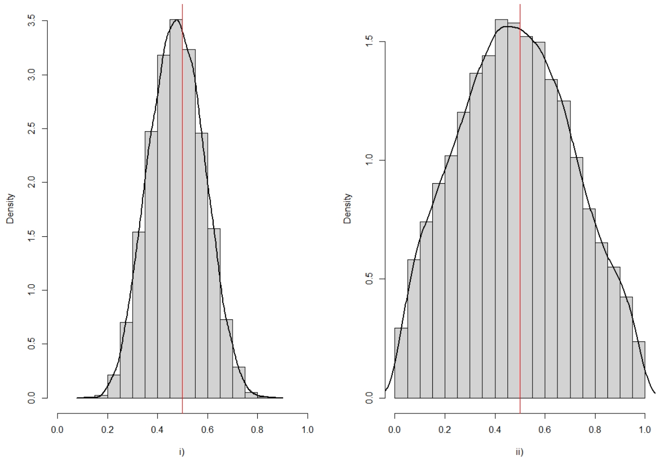

Figure 2 Prior predicted values for the i) global cure fraction and ii) end-point specific cure fractions.

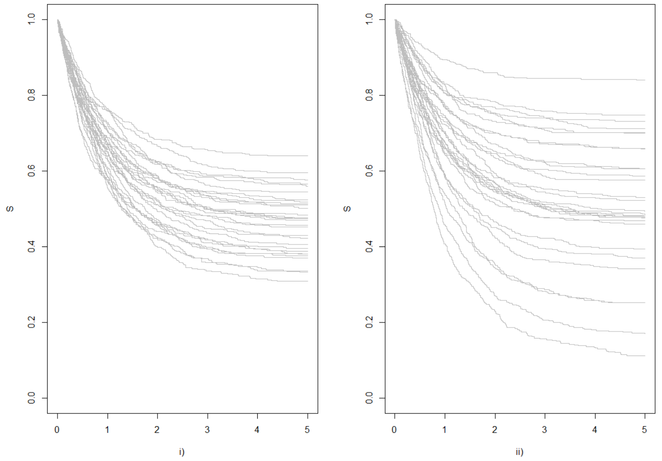

Figure 3 Kaplan-Meier curves using prior predicted values for the i) global cure fraction and ii) end-point-specific cure fractions. The underlying, latent survival distribution is Exp(1) for the uncured fraction.

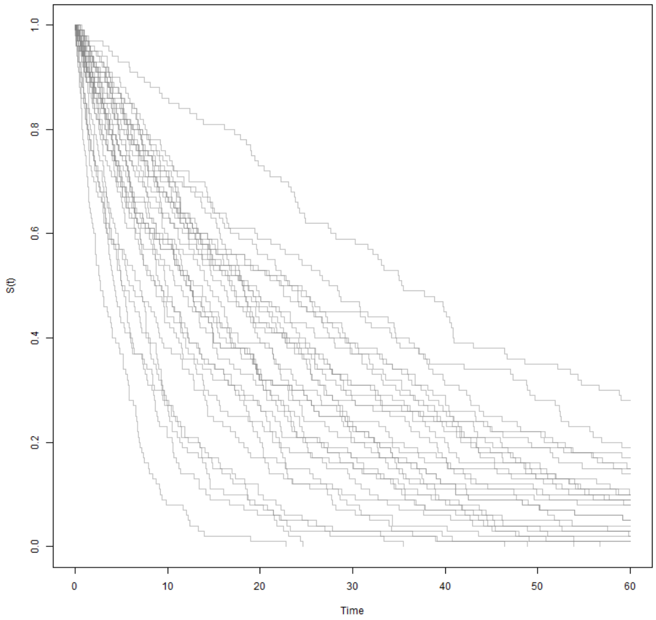

Figure 4 Kaplan-Meier curves using realisations from the latent model parameter prior distribution and an exponential survival distribution.

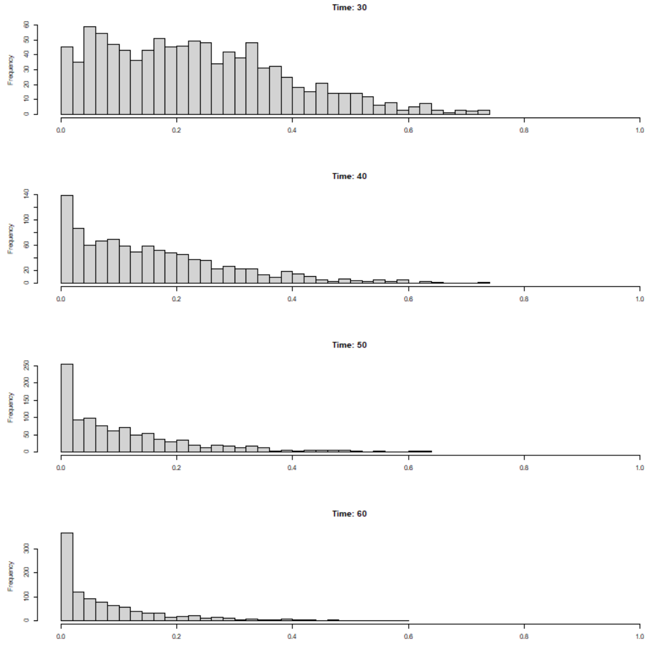

Figure 5 Histograms of survival probabilities at 30, 40, 50 and 60 months.

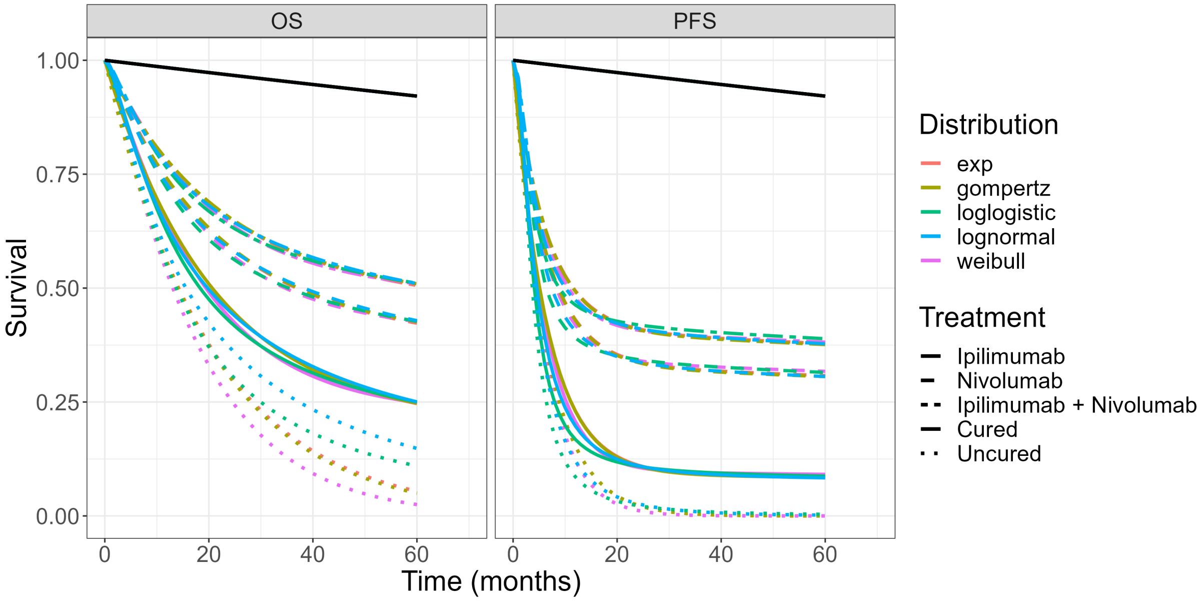

Figure 6 Bayesian hierarchical mixture cure model posterior survival curves for a range of distributions using complete CheckMate 067 trial data. The same distribution pairs are used for OS and PFS.

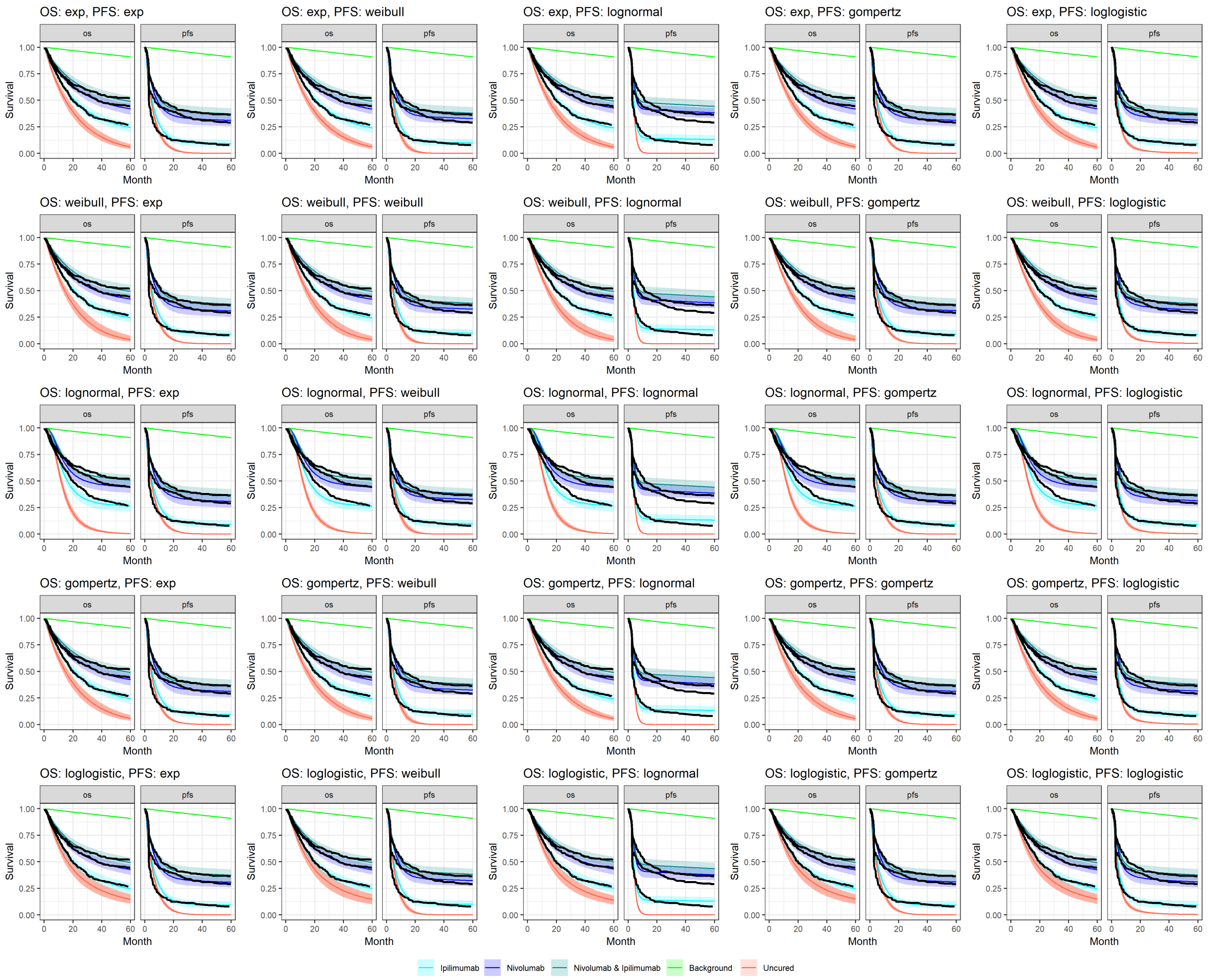

Figure 7 Hierarchical model posterior survival curves for exponential, weibull, gompertz, log-logistic and log-Normal uncured fraction for OS and PFS events and ipilimumab, nivolumab and combination treatments. The black lines show the Kaplan-Meier curves using CheckMate 067 trial data.

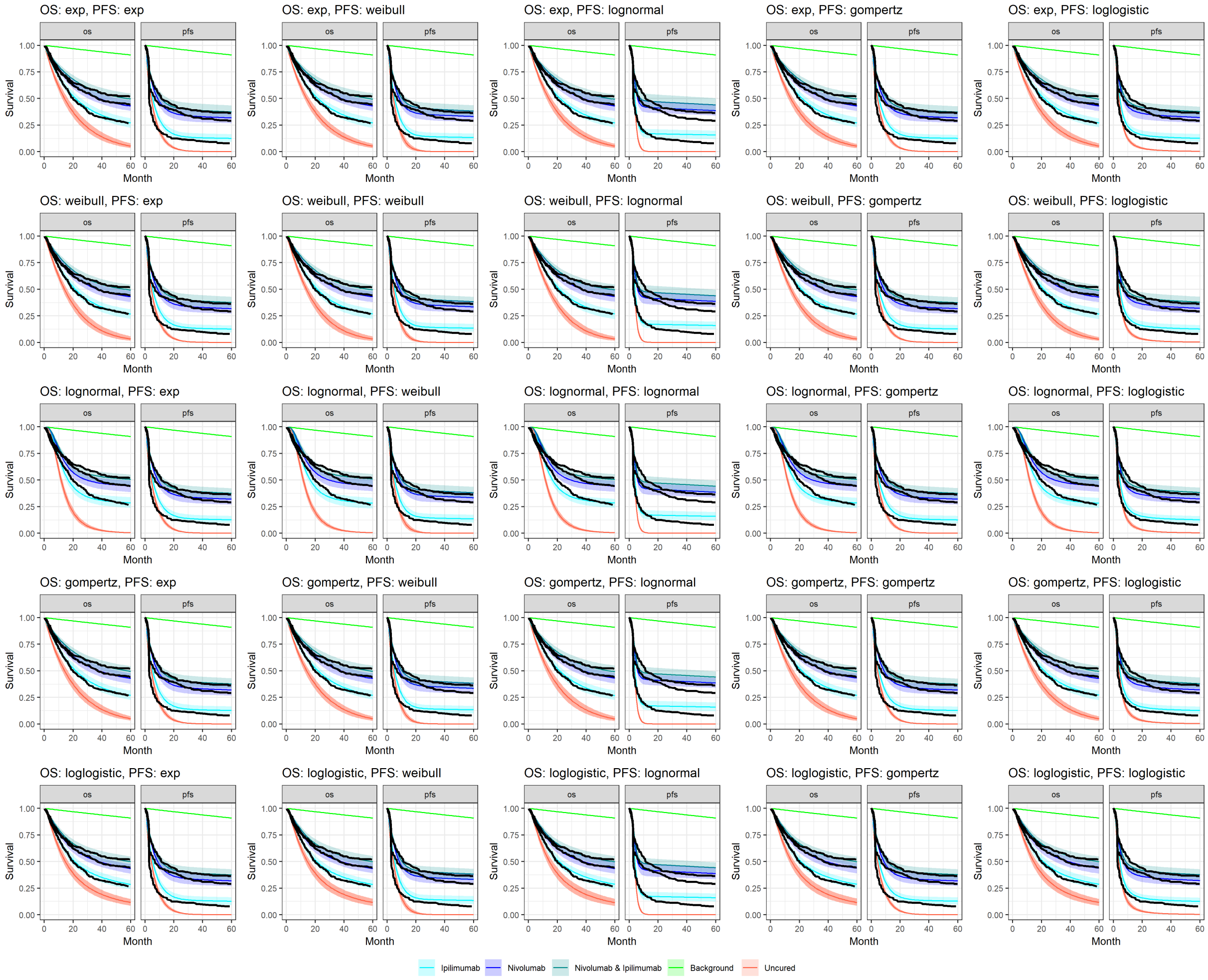

Figure 8 Separate model posterior survival curves for exponential, weibull, gompertz, log-logistic and log-Normal uncured fraction for OS and PFS events ipilimumab, nivolumab and combination treatments. The black lines show the Kaplan-Meier curves using CheckMate 067 trial data.

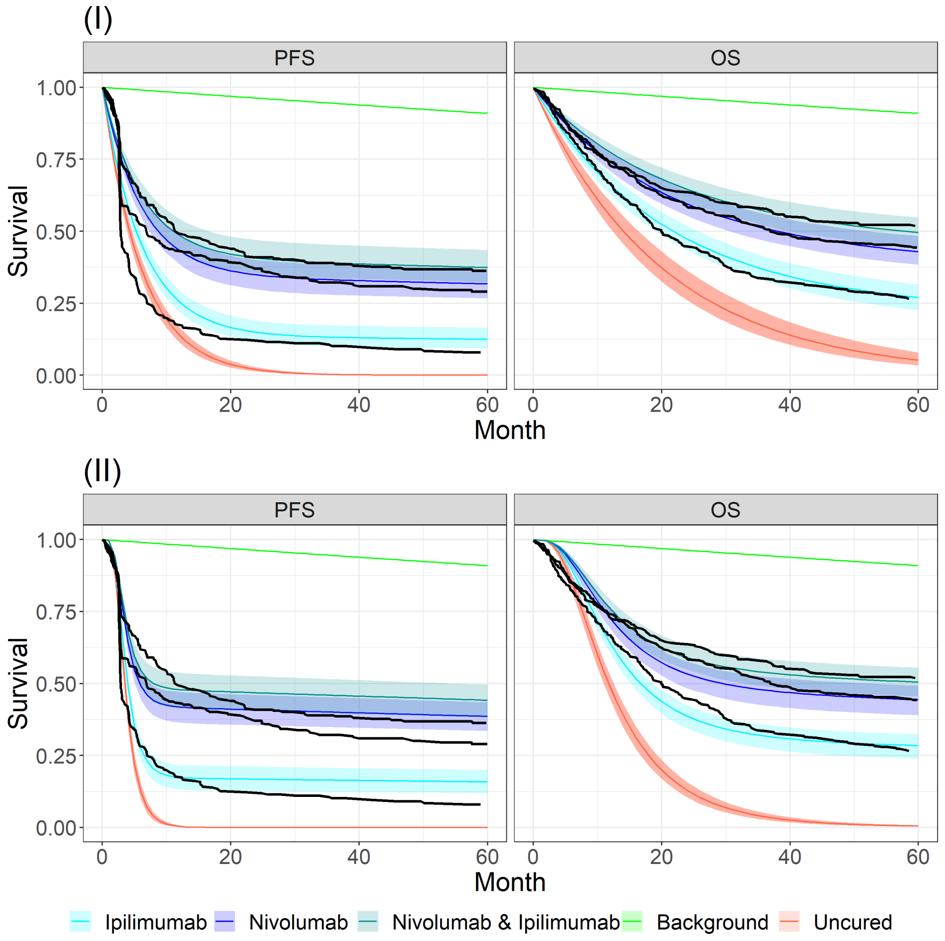

Figure 9 Separate mixture cure models posterior survival curves for uncured fraction assumed (I) exponential; and (II) log-Normal for both OS and PFS, and ipilimumab, nivolumab and combination treatments. The black lines show the Kaplan-Meier curves using the complete CheckMate 067 trial data.

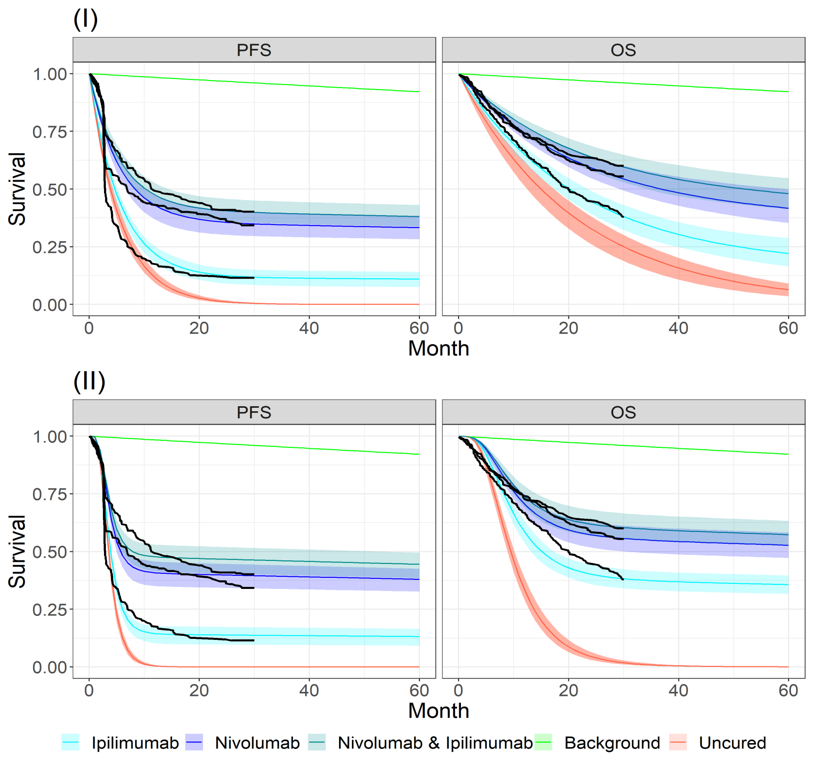

Figure 10 Separate mixture cure models posterior survival curves with 95% credible intervals for (I) PFS and OS exponential uncured survival curves and (II) PFS and OS log-Normal uncured survival curves and censored times at a 30 month cutpoint. The black lines show the observed data Kaplan-Meier curves.

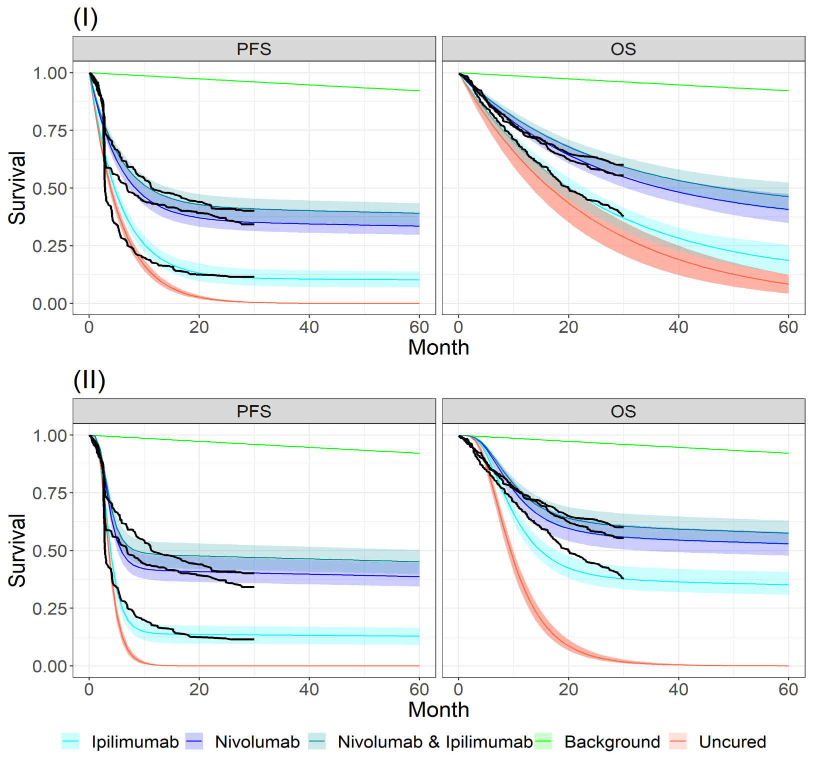

Figure 11 Hierarchical mixture cure model posterior survival curves with 95% credible intervals for (I) PFS and OS exponential uncured survival curves and (II) PFS and OS log-Normal uncured survival curves and censored times at a 30 month cutpoint. The black lines show the observed data Kaplan-Meier curves.

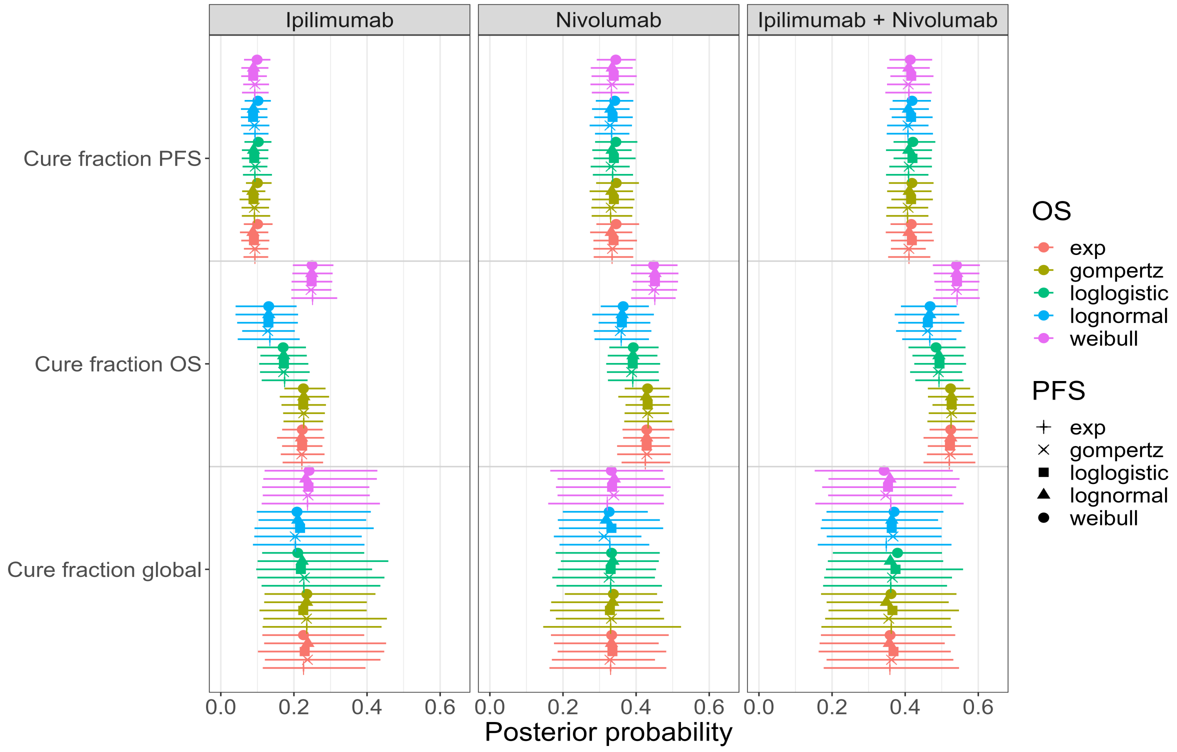

Figure 12 Hierarchical mixture cure model posterior cure fraction forest plots with 95% credible intervals for (i) ipilimumab only (ii) nivolumab only and (iii) combination treatment.

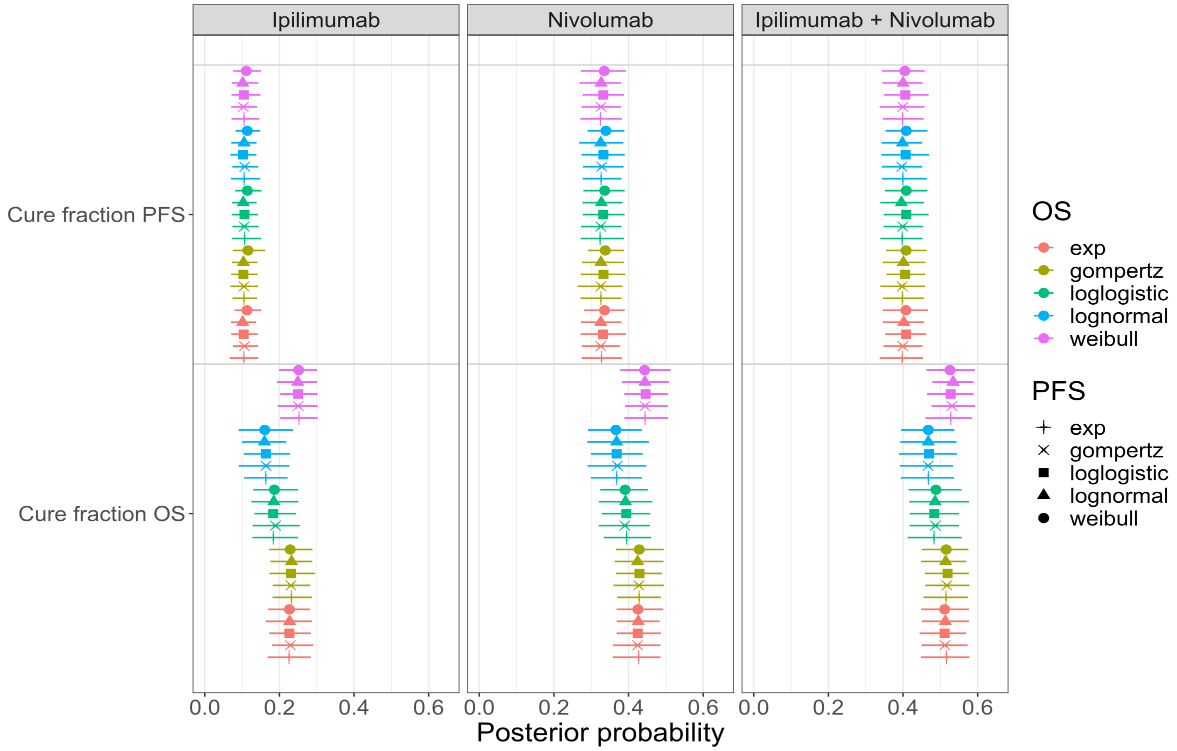

Figure 13 Separate mixture cure models posterior cure fraction forest plots with 95% credible intervals for (i) ipilimumab only (ii) nivolumab only and (iii) combination treatment.

Table 1 Posterior summary statistics for hierarchical mixture cure model parameters and all OS distributions with exponential PFS distribution.

	Exp/Exp[1]		Weibull/Exp		Gompertz/Exp		Log-logistic/Exp		log-Normal/Exp	
**Parameter**	**Mean**	**95% CrI**	**Mean**	**95% CrI**	**Mean**	**95% CrI**	**Mean**	**95% CrI**	**Mean**	**95% CrI**
$\beta_{os,0}$	-3.093	(-3.249, -2.956)	3.032	(2.903, 3.221)	-3.094	(-3.264, -2.948)	2.831	(2.659, 3.023)	2.478	(2.406, 2.559)
$\beta_{os,1}$	-0.014	(-0.023, -0.005)	0.011	(0.003, 0.021)	-0.014	(-0.024, -0.003)	0.011	(0, 0.021)	0.001	(-0.004, 0.006)
$\beta_{pfs,0}$	-1.825	(-1.939, -1.728)	-1.828	(-1.925, -1.738)	-1.827	(-1.92, -1.73)	-1.831	(-1.921, -1.734)	-1.831	(-1.924, -1.732)
$\beta_{pfs,1}$	-0.003	(-0.009, 0.004)	-0.003	(-0.009, 0.003)	-0.003	(-0.01, 0.003)	-0.003	(-0.009, 0.003)	-0.003	(-0.009, 0.004)
β_1^{π}	-1.179	(-2.283, 0.153)	-1.102	(-2.009, 0.28)	-1.168	(-2.115, 0.18)	-1.447	(-2.351, 0.022)	-1.005	(-1.966, 0.386)
β_2^{π}	-0.527	(-1.604, 0.351)	-0.494	(-1.337, 0.485)	-0.496	(-1.4, 0.341)	-0.625	(-1.509, 0.231)	-0.463	(-1.565, 0.498)
β_3^{π}	-0.311	(-1.382, 0.508)	-0.324	(-1.392, 0.62)	-0.348	(-1.422, 0.565)	-0.354	(-1.35, 0.318)	-0.228	(-1.269, 0.706)
σ_1	1.403	(0.339, 3.87)	1.486	(0.325, 4.266)	1.542	(0.302, 4.395)	1.268	(0.058, 4.274)	1.514	(0.399, 3.907)
σ_2	0.791	(0.056, 2.834)	0.868	(0.111, 3.433)	0.846	(0.064, 2.911)	0.701	(0.012, 2.897)	1.132	(0.197, 4.121)
σ_3	0.852	(0.091, 2.636)	0.985	(0.146, 3.444)	1.008	(0.116, 3.424)	0.703	(0.016, 2.906)	1.102	(0.213, 3.506)
$\pi_{global,1}$	0.252	(0.093, 0.538)	0.265	(0.118, 0.57)	0.253	(0.108, 0.545)	0.209	(0.087, 0.505)	0.282	(0.123, 0.595)
$\pi_{global,2}$	0.377	(0.167, 0.587)	0.384	(0.208, 0.619)	0.384	(0.198, 0.584)	0.354	(0.181, 0.557)	0.393	(0.173, 0.622)
$\pi_{global,3}$	0.427	(0.201, 0.624)	0.425	(0.199, 0.65)	0.420	(0.194, 0.638)	0.416	(0.206, 0.579)	0.447	(0.219, 0.67)
$\pi_{os,1}$	0.216	(0.158, 0.275)	0.238	(0.181, 0.302)	0.219	(0.156, 0.278)	0.130	(0.033, 0.211)	0.286	(0.228, 0.344)
$\pi_{os,2}$	0.423	(0.359, 0.484)	0.440	(0.375, 0.499)	0.429	(0.364, 0.494)	0.366	(0.291, 0.447)	0.483	(0.426, 0.546)
$\pi_{os,3}$	0.505	(0.437, 0.569)	0.523	(0.45, 0.593)	0.508	(0.439, 0.569)	0.455	(0.388, 0.524)	0.557	(0.503, 0.611)
$\pi_{pfs,1}$	0.101	(0.068, 0.139)	0.099	(0.065, 0.135)	0.100	(0.065, 0.137)	0.102	(0.068, 0.137)	0.099	(0.067, 0.142)
$\pi_{pfs,2}$	0.341	(0.282, 0.404)	0.340	(0.281, 0.401)	0.342	(0.284, 0.398)	0.338	(0.285, 0.386)	0.338	(0.276, 0.4)
$\pi_{pfs,3}$	0.409	(0.351, 0.468)	0.410	(0.353, 0.472)	0.407	(0.351, 0.466)	0.411	(0.345, 0.472)	0.407	(0.348, 0.467)

1OS distribution/PFS distribution.

Table 2 Posterior summary statistics for hierarchical mixture cure model parameters and all OS distributions with weibull PFS distribution.

	Exp/Weibull[1]		Weibull/Weibull		Gompertz/Weibull		Log-logistic/Weibull		log-Normal/Weibull	
**Parameter**	**Mean**	**95% CrI**	**Mean**	**95% CrI**	**Mean**	**95% CrI**	**Mean**	**95% CrI**	**Mean**	**95% CrI**
$\beta_{os,0}$	-3.097	(-3.247, -2.968)	3.032	(2.894, 3.198)	-3.092	(-3.243, -2.949)	2.826	(2.661, 3.011)	2.478	(2.408, 2.548)
$\beta_{os,1}$	-0.014	(-0.023, -0.005)	0.011	(0.002, 0.02)	-0.013	(-0.023, -0.004)	0.011	(0.002, 0.022)	0.000	(-0.004, 0.005)
$\beta_{pfs,0}$	1.799	(1.705, 1.899)	1.797	(1.7, 1.899)	1.798	(1.711, 1.904)	1.802	(1.711, 1.901)	1.795	(1.696, 1.903)
$\beta_{pfs,1}$	0.002	(-0.004, 0.007)	0.001	(-0.005, 0.007)	0.001	(-0.005, 0.008)	0.002	(-0.004, 0.007)	0.001	(-0.005, 0.008)
β_1^{π}	-1.208	(-2.243, 0.204)	-1.056	(-2.104, 0.188)	-1.159	(-2.094, 0.307)	-1.450	(-2.403, 0.021)	-1.035	(-2.091, 0.319)
β_2^{π}	-0.518	(-1.498, 0.286)	-0.482	(-1.445, 0.439)	-0.487	(-1.319, 0.3)	-0.612	(-1.554, -0.001)	-0.393	(-1.525, 0.595)
β_3^{π}	-0.306	(-1.473, 0.563)	-0.324	(-1.51, 0.557)	-0.259	(-1.334, 0.428)	-0.340	(-1.353, 0.327)	-0.239	(-1.429, 0.632)
σ_1	1.359	(0.223, 4.202)	1.485	(0.267, 4.21)	1.335	(0.247, 3.67)	1.089	(0.036, 3.606)	1.568	(0.389, 3.867)
σ_2	0.764	(0.026, 3.09)	0.851	(0.071, 3.113)	0.704	(0.033, 2.6)	0.639	(0.015, 2.502)	0.984	(0.174, 3.182)
σ_3	0.888	(0.09, 2.978)	1.081	(0.125, 3.455)	0.825	(0.09, 2.732)	0.572	(0.01, 2.264)	1.032	(0.179, 3.115)
$\pi_{global,1}$	0.247	(0.096, 0.551)	0.273	(0.109, 0.547)	0.255	(0.11, 0.576)	0.209	(0.083, 0.505)	0.277	(0.11, 0.579)
$\pi_{global,2}$	0.378	(0.183, 0.571)	0.387	(0.191, 0.608)	0.385	(0.211, 0.574)	0.356	(0.175, 0.5)	0.409	(0.179, 0.644)
$\pi_{global,3}$	0.429	(0.186, 0.637)	0.426	(0.181, 0.636)	0.440	(0.209, 0.605)	0.419	(0.205, 0.581)	0.445	(0.193, 0.653)
$\pi_{os,1}$	0.211	(0.147, 0.266)	0.236	(0.177, 0.296)	0.219	(0.156, 0.281)	0.134	(0.049, 0.222)	0.285	(0.232, 0.331)
$\pi_{os,2}$	0.421	(0.359, 0.482)	0.438	(0.369, 0.501)	0.426	(0.356, 0.49)	0.366	(0.3, 0.437)	0.483	(0.429, 0.538)
$\pi_{os,3}$	0.506	(0.436, 0.572)	0.522	(0.456, 0.58)	0.511	(0.438, 0.567)	0.454	(0.377, 0.532)	0.557	(0.489, 0.615)
$\pi_{pfs,1}$	0.108	(0.072, 0.146)	0.109	(0.077, 0.15)	0.109	(0.073, 0.156)	0.109	(0.076, 0.147)	0.108	(0.069, 0.146)
$\pi_{pfs,2}$	0.359	(0.304, 0.417)	0.358	(0.301, 0.414)	0.356	(0.301, 0.413)	0.354	(0.297, 0.409)	0.359	(0.301, 0.424)
$\pi_{pfs,3}$	0.416	(0.358, 0.479)	0.419	(0.36, 0.476)	0.417	(0.356, 0.477)	0.420	(0.361, 0.478)	0.415	(0.363, 0.472)

1OS distribution/PFS distribution.

Table 3 Posterior summary statistics for hierarchical mixture cure model parameters and all OS distributions with gompertz PFS distribution.

	Exp/Gompertz[1]		Weibull/Gompertz		Gompertz/Gompertz		Log-logistic/Gompertz		log-Normal/Gompertz	
**Parameter**	**Mean**	**95% CrI**	**Mean**	**95% CrI**	**Mean**	**95% CrI**	**Mean**	**95% CrI**	**Mean**	**95% CrI**
$\beta_{os,0}$	-3.094	(-3.247, -2.956)	3.036	(2.922, 3.196)	-3.091	(-3.27, -2.944)	2.844	(2.671, 3.03)	2.477	(2.41, 2.553)
$\beta_{os,1}$	-0.014	(-0.023, -0.005)	0.011	(0.002, 0.02)	-0.013	(-0.023, -0.004)	0.011	(0.002, 0.021)	0.001	(-0.004, 0.006)
$\beta_{pfs,0}$	-1.827	(-1.929, -1.727)	-1.834	(-1.933, -1.729)	-1.831	(-1.947, -1.721)	-1.828	(-1.92, -1.726)	-1.828	(-1.933, -1.729)
$\beta_{pfs,1}$	-0.003	(-0.01, 0.004)	-0.003	(-0.009, 0.004)	-0.003	(-0.009, 0.003)	-0.003	(-0.01, 0.003)	-0.003	(-0.009, 0.004)
β_1^{π}	-1.171	(-2.273, 0.111)	-1.120	(-2.117, 0.125)	-1.105	(-2.281, 0.272)	-1.465	(-2.526, 0.364)	-1.017	(-2.152, 0.291)
β_2^{π}	-0.521	(-1.482, 0.441)	-0.526	(-1.562, 0.259)	-0.490	(-1.468, 0.38)	-0.579	(-1.427, 0.235)	-0.478	(-1.687, 0.523)
β_3^{π}	-0.350	(-1.62, 0.563)	-0.311	(-1.36, 0.785)	-0.299	(-1.299, 0.579)	-0.375	(-1.256, 0.46)	-0.214	(-1.539, 0.759)
σ_1	1.362	(0.3, 4.001)	1.481	(0.38, 4.043)	1.548	(0.324, 3.606)	1.142	(0.034, 3.456)	1.640	(0.463, 4.084)
σ_2	0.810	(0.06, 3.291)	0.960	(0.092, 3.207)	0.933	(0.088, 3.125)	0.607	(0.013, 2.766)	1.070	(0.218, 3.189)
σ_3	0.973	(0.081, 3.63)	0.990	(0.151, 3.144)	0.912	(0.07, 3.307)	0.692	(0.033, 3.032)	1.068	(0.176, 3.85)
$\pi_{global,1}$	0.254	(0.093, 0.528)	0.262	(0.107, 0.531)	0.267	(0.093, 0.568)	0.208	(0.074, 0.59)	0.281	(0.104, 0.572)
$\pi_{global,2}$	0.378	(0.185, 0.608)	0.377	(0.173, 0.564)	0.386	(0.187, 0.594)	0.363	(0.194, 0.559)	0.390	(0.156, 0.628)
$\pi_{global,3}$	0.420	(0.165, 0.637)	0.428	(0.204, 0.687)	0.429	(0.214, 0.641)	0.411	(0.222, 0.613)	0.452	(0.177, 0.681)
$\pi_{os,1}$	0.214	(0.148, 0.282)	0.235	(0.169, 0.304)	0.219	(0.163, 0.275)	0.128	(0.051, 0.208)	0.287	(0.232, 0.355)
$\pi_{os,2}$	0.421	(0.352, 0.485)	0.439	(0.372, 0.509)	0.426	(0.36, 0.492)	0.363	(0.287, 0.436)	0.485	(0.425, 0.551)
$\pi_{os,3}$	0.507	(0.441, 0.576)	0.522	(0.453, 0.588)	0.507	(0.444, 0.571)	0.452	(0.378, 0.521)	0.554	(0.497, 0.618)
$\pi_{pfs,1}$	0.101	(0.063, 0.139)	0.099	(0.066, 0.14)	0.100	(0.066, 0.137)	0.100	(0.068, 0.139)	0.100	(0.065, 0.137)
$\pi_{pfs,2}$	0.341	(0.282, 0.4)	0.342	(0.282, 0.401)	0.343	(0.283, 0.397)	0.341	(0.286, 0.394)	0.341	(0.287, 0.398)
$\pi_{pfs,3}$	0.411	(0.35, 0.471)	0.408	(0.349, 0.469)	0.410	(0.351, 0.47)	0.404	(0.349, 0.462)	0.410	(0.346, 0.472)

1OS distribution/PFS distribution.

Table 4 Posterior summary statistics for hierarchical mixture cure model parameters and all OS distributions with log-logistic PFS distribution.

	**Exp/Log-logistic**[1]		**Weibull/Log-logistic**		**Gompertz/Log-logistic**		**Log-logistic/Log-logistic**		**log-Normal/Log-logistic**	
**Parameter**	**Mean**	**95% CrI**	**Mean**	**95% CrI**	**Mean**	**95% CrI**	**Mean**	**95% CrI**	**Mean**	**95% CrI**
$\beta_{os,0}$	-3.093	(-3.258, -2.954)	3.025	(2.915, 3.185)	-3.090	(-3.241, -2.923)	2.842	(2.659, 3.034)	2.478	(2.419, 2.556)
$\beta_{os,1}$	-0.014	(-0.022, -0.005)	0.011	(0.003, 0.02)	-0.013	(-0.023, -0.004)	0.011	(0.002, 0.021)	0.000	(-0.005, 0.006)
$\beta_{pfs,0}$	1.334	(1.257, 1.41)	1.337	(1.269, 1.406)	1.334	(1.264, 1.406)	1.340	(1.259, 1.413)	1.336	(1.272, 1.406)
$\beta_{pfs,1}$	0.002	(-0.002, 0.007)	0.002	(-0.002, 0.008)	0.002	(-0.002, 0.007)	0.002	(-0.002, 0.007)	0.003	(-0.002, 0.008)
β_1^{π}	-1.170	(-2.267, 0.343)	-1.085	(-2.176, 0.456)	-1.209	(-2.253, 0.164)	-1.440	(-2.431, 0.204)	-0.972	(-2.091, 0.444)
β_2^{π}	-0.529	(-1.444, 0.278)	-0.508	(-1.428, 0.449)	-0.506	(-1.671, 0.614)	-0.611	(-1.555, 0.235)	-0.417	(-1.429, 0.442)
β_3^{π}	-0.302	(-1.419, 0.533)	-0.292	(-1.407, 0.578)	-0.265	(-1.314, 0.505)	-0.355	(-1.209, 0.372)	-0.261	(-1.31, 0.625)
σ_1	1.535	(0.312, 4.338)	1.632	(0.381, 4.085)	1.517	(0.368, 4.542)	1.311	(0.065, 3.968)	1.662	(0.572, 4.064)
σ_2	0.883	(0.045, 3.188)	0.831	(0.048, 2.777)	0.924	(0.037, 3.221)	0.646	(0.01, 2.516)	1.015	(0.169, 3.317)
σ_3	0.902	(0.07, 2.804)	0.998	(0.152, 3.23)	0.883	(0.092, 3.001)	0.674	(0.024, 2.829)	1.074	(0.209, 3.007)
$\pi_{global,1}$	0.255	(0.094, 0.585)	0.270	(0.102, 0.612)	0.247	(0.095, 0.541)	0.213	(0.081, 0.551)	0.290	(0.11, 0.609)
$\pi_{global,2}$	0.376	(0.191, 0.569)	0.381	(0.193, 0.61)	0.382	(0.158, 0.649)	0.357	(0.174, 0.559)	0.402	(0.193, 0.609)
$\pi_{global,3}$	0.430	(0.195, 0.63)	0.432	(0.197, 0.641)	0.438	(0.212, 0.624)	0.415	(0.23, 0.592)	0.439	(0.212, 0.651)
$\pi_{os,1}$	0.214	(0.153, 0.276)	0.239	(0.181, 0.304)	0.220	(0.159, 0.285)	0.127	(0.044, 0.206)	0.290	(0.234, 0.345)
$\pi_{os,2}$	0.423	(0.354, 0.493)	0.438	(0.369, 0.498)	0.428	(0.365, 0.492)	0.362	(0.291, 0.432)	0.482	(0.431, 0.537)
$\pi_{os,3}$	0.508	(0.445, 0.568)	0.523	(0.456, 0.589)	0.510	(0.443, 0.572)	0.451	(0.381, 0.531)	0.554	(0.495, 0.619)
$\pi_{pfs,1}$	0.095	(0.062, 0.142)	0.091	(0.061, 0.13)	0.094	(0.061, 0.135)	0.096	(0.06, 0.139)	0.093	(0.061, 0.136)
$\pi_{pfs,2}$	0.343	(0.282, 0.402)	0.347	(0.292, 0.413)	0.344	(0.287, 0.406)	0.339	(0.28, 0.398)	0.343	(0.283, 0.407)
$\pi_{pfs,3}$	0.412	(0.35, 0.47)	0.412	(0.354, 0.472)	0.414	(0.359, 0.47)	0.410	(0.354, 0.472)	0.409	(0.349, 0.462)

1OS distribution/PFS distribution.

Table 5 Posterior summary statistics for hierarchical mixture cure model parameters and all OS distributions with log-Normal PFS distribution.

	Exp/log-Normal[1]		Weibull/Log-Normal		Gompertz/Log-Normal		Log-logistic/Log-Normal		log-Normal/Log-Normal	
**Parameter**	**Mean**	**95% CrI**	**Mean**	**95% CrI**	**Mean**	**95% CrI**	**Mean**	**95% CrI**	**Mean**	**95% CrI**
$\beta_{os,0}$	-3.085	(-3.248, -2.943)	3.027	(2.893, 3.19)	-3.086	(-3.244, -2.956)	2.802	(2.646, 2.985)	2.481	(2.399, 2.551)
$\beta_{os,1}$	-0.014	(-0.023, -0.005)	0.011	(0.003, 0.02)	-0.013	(-0.022, -0.005)	0.010	(0, 0.019)	0.000	(-0.004, 0.005)
$\beta_{pfs,0}$	1.250	(1.206, 1.297)	1.249	(1.204, 1.301)	1.251	(1.205, 1.299)	1.252	(1.205, 1.299)	1.248	(1.202, 1.297)
$\beta_{pfs,1}$	0.000	(-0.003, 0.003)	0.000	(-0.003, 0.003)	0.000	(-0.004, 0.004)	0.000	(-0.003, 0.003)	0.000	(-0.003, 0.003)
β_1^{π}	-1.134	(-1.973, 0.179)	-1.133	(-2.027, 0.034)	-1.175	(-2.171, 0.071)	-1.383	(-2.181, 0.117)	-0.976	(-1.875, 0.358)
β_2^{π}	-0.379	(-1.346, 0.166)	-0.338	(-1.213, 0.314)	-0.366	(-1.339, 0.281)	-0.457	(-1.326, 0.208)	-0.313	(-1.075, 0.281)
β_3^{π}	-0.109	(-1.035, 0.607)	-0.112	(-1.109, 0.525)	-0.104	(-1.075, 0.625)	-0.179	(-1.153, 0.438)	-0.123	(-1.113, 0.646)
σ_1	1.114	(0.131, 3.274)	1.167	(0.17, 3.696)	1.141	(0.095, 3.668)	0.885	(0.017, 3.264)	1.242	(0.292, 4.03)
σ_2	0.603	(0.013, 2.647)	0.570	(0.015, 2.431)	0.548	(0.011, 2.397)	0.586	(0.011, 2.643)	0.719	(0.026, 3.029)
σ_3	0.629	(0.015, 2.941)	0.677	(0.034, 3.101)	0.569	(0.016, 2.282)	0.571	(0.008, 2.638)	0.763	(0.048, 2.756)
$\pi_{global,1}$	0.256	(0.122, 0.545)	0.256	(0.116, 0.508)	0.250	(0.102, 0.518)	0.215	(0.101, 0.529)	0.285	(0.133, 0.589)
$\pi_{global,2}$	0.410	(0.206, 0.541)	0.419	(0.229, 0.578)	0.413	(0.208, 0.57)	0.391	(0.21, 0.552)	0.425	(0.254, 0.57)
$\pi_{global,3}$	0.475	(0.262, 0.647)	0.474	(0.248, 0.628)	0.476	(0.254, 0.651)	0.457	(0.24, 0.608)	0.472	(0.247, 0.656)
$\pi_{os,1}$	0.212	(0.15, 0.273)	0.236	(0.172, 0.296)	0.216	(0.156, 0.282)	0.145	(0.069, 0.217)	0.288	(0.231, 0.342)
$\pi_{os,2}$	0.433	(0.375, 0.489)	0.440	(0.381, 0.502)	0.429	(0.367, 0.487)	0.382	(0.3, 0.456)	0.479	(0.42, 0.543)
$\pi_{os,3}$	0.509	(0.449, 0.567)	0.521	(0.458, 0.583)	0.509	(0.451, 0.565)	0.468	(0.396, 0.539)	0.554	(0.5, 0.607)
$\pi_{pfs,1}$	0.143	(0.101, 0.189)	0.144	(0.098, 0.188)	0.145	(0.105, 0.19)	0.141	(0.103, 0.184)	0.145	(0.103, 0.193)
$\pi_{pfs,2}$	0.420	(0.364, 0.469)	0.423	(0.37, 0.478)	0.420	(0.361, 0.479)	0.414	(0.356, 0.472)	0.424	(0.365, 0.48)
$\pi_{pfs,3}$	0.487	(0.426, 0.545)	0.486	(0.43, 0.549)	0.485	(0.427, 0.543)	0.478	(0.419, 0.538)	0.486	(0.424, 0.545)

1OS distribution/PFS distribution.

Table 6 Posterior summary statistics for separate mixture cure models parameters and all OS distributions with exponential PFS distribution.

	Exp/Exp[1]		Weibull/Exp		Gompertz/Exp		Log-logistic/Exp		log-Normal/Exp	
**Parameter**	**Mean**	**95% CrI**	**Mean**	**95% CrI**	**Mean**	**95% CrI**	**Mean**	**95% CrI**	**Mean**	**95% CrI**
β_0^{os}	-3.042	(-3.203, -2.909)	3.002	(2.886, 3.141)	-3.051	(-3.197, -2.914)	2.715	(2.583, 2.858)	2.472	(2.392, 2.547)
β_1^{os}	-0.012	(-0.021, -0.003)	0.009	(0.001, 0.018)	-0.012	(-0.021, -0.003)	0.007	(-0.001, 0.016)	0.000	(-0.004, 0.005)
β_0^{pfs}	-1.817	(-1.915, -1.719)	-1.815	(-1.917, -1.728)	-1.816	(-1.929, -1.712)	-1.817	(-1.919, -1.718)	-1.815	(-1.91, -1.719)
β_1^{pfs}	-0.003	(-0.009, 0.004)	-0.002	(-0.009, 0.004)	-0.002	(-0.008, 0.005)	-0.003	(-0.009, 0.003)	-0.003	(-0.009, 0.003)
$\beta_{os,1}^{\pi}$	-1.084	(-1.399, -0.816)	-1.008	(-1.32, -0.756)	-1.077	(-1.39, -0.787)	-1.308	(-1.699, -0.999)	-0.805	(-1.05, -0.528)
$\beta_{os,2}^{\pi}$	-0.245	(-0.488, 0.014)	-0.190	(-0.429, 0.08)	-0.238	(-0.486, 0.017)	-0.398	(-0.715, -0.15)	-0.057	(-0.323, 0.168)
$\beta_{os,3}^{\pi}$	0.068	(-0.185, 0.318)	0.102	(-0.125, 0.344)	0.070	(-0.183, 0.324)	-0.066	(-0.382, 0.196)	0.218	(-0.014, 0.425)
$\beta_{pfs,1}^{\pi}$	-1.848	(-2.174, -1.508)	-1.839	(-2.158, -1.542)	-1.829	(-2.171, -1.502)	-1.831	(-2.191, -1.507)	-1.836	(-2.128, -1.525)
$\beta_{pfs,2}^{\pi}$	-0.626	(-0.878, -0.385)	-0.625	(-0.86, -0.398)	-0.617	(-0.881, -0.36)	-0.622	(-0.872, -0.372)	-0.613	(-0.869, -0.402)
$\beta_{pfs,3}^{\pi}$	-0.361	(-0.595, -0.088)	-0.353	(-0.582, -0.11)	-0.348	(-0.545, -0.11)	-0.359	(-0.59, -0.117)	-0.353	(-0.591, -0.141)
π_1^{os}	0.254	(0.198, 0.307)	0.268	(0.211, 0.32)	0.255	(0.199, 0.313)	0.214	(0.155, 0.269)	0.310	(0.259, 0.371)
π_2^{os}	0.439	(0.38, 0.504)	0.453	(0.394, 0.52)	0.441	(0.381, 0.504)	0.402	(0.328, 0.463)	0.486	(0.42, 0.542)
π_3^{os}	0.517	(0.454, 0.579)	0.525	(0.469, 0.585)	0.518	(0.454, 0.58)	0.484	(0.406, 0.549)	0.554	(0.497, 0.605)
π_1^{pfs}	0.137	(0.102, 0.181)	0.138	(0.104, 0.176)	0.140	(0.102, 0.182)	0.139	(0.101, 0.181)	0.139	(0.106, 0.179)
π_2^{pfs}	0.349	(0.294, 0.405)	0.349	(0.297, 0.402)	0.351	(0.293, 0.411)	0.350	(0.295, 0.408)	0.352	(0.296, 0.401)
π_3^{pfs}	0.411	(0.355, 0.478)	0.413	(0.358, 0.472)	0.414	(0.367, 0.473)	0.411	(0.357, 0.471)	0.413	(0.356, 0.465)

1OS distribution/PFS distribution.

Table 7 Posterior summary statistics for separate mixture cure models parameters and all OS distributions with weibull PFS distribution.

	**Exp/Weibull**[1]		**Weibull/Weibull**		**Gompertz/Weibull**		**Log-logistic/Weibull**		**log-Normal/Weibull**	
**Parameter**	**Mean**	**95% CrI**	**Mean**	**95% CrI**	**Mean**	**95% CrI**	**Mean**	**95% CrI**	**Mean**	**95% CrI**
β_0^{os}	-3.040	(-3.18, -2.913)	2.996	(2.877, 3.125)	-3.052	(-3.182, -2.914)	2.717	(2.585, 2.853)	2.474	(2.398, 2.546)
β_1^{os}	-0.012	(-0.022, -0.003)	0.009	(0.001, 0.018)	-0.012	(-0.022, -0.003)	0.008	(0, 0.016)	0.000	(-0.005, 0.006)
β_0^{pfs}	1.780	(1.682, 1.887)	1.778	(1.683, 1.864)	1.774	(1.675, 1.869)	1.775	(1.683, 1.87)	1.775	(1.676, 1.87)
β_1^{pfs}	0.001	(-0.005, 0.007)	0.000	(-0.006, 0.006)	0.001	(-0.005, 0.007)	0.000	(-0.004, 0.006)	0.001	(-0.006, 0.007)
$\beta_{os,1}^{\pi}$	-1.075	(-1.395, -0.786)	-1.004	(-1.315, -0.736)	-1.068	(-1.364, -0.792)	-1.309	(-1.659, -0.978)	-0.817	(-1.085, -0.56)
$\beta_{os,2}^{\pi}$	-0.239	(-0.475, 0.005)	-0.188	(-0.43, 0.038)	-0.244	(-0.495, 0.004)	-0.401	(-0.711, -0.101)	-0.048	(-0.276, 0.202)
$\beta_{os,3}^{\pi}$	0.062	(-0.196, 0.335)	0.107	(-0.18, 0.34)	0.071	(-0.184, 0.343)	-0.069	(-0.325, 0.218)	0.236	(0.009, 0.448)
$\beta_{pfs,1}^{\pi}$	-1.772	(-2.083, -1.467)	-1.763	(-2.077, -1.437)	-1.770	(-2.116, -1.473)	-1.762	(-2.084, -1.432)	-1.754	(-2.09, -1.445)
$\beta_{pfs,2}^{\pi}$	-0.562	(-0.813, -0.31)	-0.545	(-0.806, -0.301)	-0.543	(-0.791, -0.301)	-0.558	(-0.799, -0.312)	-0.548	(-0.81, -0.316)
$\beta_{pfs,3}^{\pi}$	-0.323	(-0.562, -0.081)	-0.319	(-0.541, -0.115)	-0.319	(-0.557, -0.077)	-0.323	(-0.586, -0.102)	-0.330	(-0.553, -0.082)
π_1^{os}	0.256	(0.199, 0.313)	0.269	(0.212, 0.324)	0.257	(0.204, 0.312)	0.214	(0.16, 0.273)	0.307	(0.253, 0.363)
π_2^{os}	0.441	(0.383, 0.501)	0.453	(0.394, 0.51)	0.439	(0.379, 0.501)	0.402	(0.329, 0.475)	0.488	(0.431, 0.55)
π_3^{os}	0.515	(0.451, 0.583)	0.527	(0.455, 0.584)	0.518	(0.454, 0.585)	0.483	(0.419, 0.554)	0.559	(0.502, 0.61)
π_1^{pfs}	0.146	(0.111, 0.187)	0.148	(0.111, 0.192)	0.147	(0.108, 0.187)	0.148	(0.111, 0.193)	0.149	(0.11, 0.191)
π_2^{pfs}	0.364	(0.307, 0.423)	0.368	(0.309, 0.425)	0.368	(0.312, 0.425)	0.365	(0.31, 0.423)	0.367	(0.308, 0.422)
π_3^{pfs}	0.420	(0.363, 0.48)	0.421	(0.368, 0.471)	0.421	(0.364, 0.481)	0.420	(0.357, 0.474)	0.419	(0.365, 0.479)

1OS distribution/PFS distribution.

Table 8 Posterior summary statistics for separate mixture cure models parameters and all OS distributions with Gompertz PFS distribution.

	Exp/Gompertz[1]		Weibull/Gompertz		Gompertz/Gompertz		Log-logistic/Gompertz		log-Normal/Gompertz	
**Parameter**	**Mean**	**95% CrI**	**Mean**	**95% CrI**	**Mean**	**95% CrI**	**Mean**	**95% CrI**	**Mean**	**95% CrI**
β_0^{os}	-3.039	(-3.171, -2.896)	2.998	(2.887, 3.118)	-3.043	(-3.196, -2.915)	2.716	(2.582, 2.854)	2.475	(2.413, 2.546)
β_1^{os}	-0.012	(-0.02, -0.003)	0.009	(0.001, 0.018)	-0.011	(-0.02, -0.001)	0.007	(-0.002, 0.016)	0.000	(-0.005, 0.004)
β_0^{pfs}	-1.815	(-1.903, -1.707)	-1.818	(-1.915, -1.721)	-1.814	(-1.914, -1.713)	-1.815	(-1.914, -1.725)	-1.817	(-1.913, -1.713)
β_1^{pfs}	-0.003	(-0.009, 0.004)	-0.003	(-0.009, 0.004)	-0.002	(-0.009, 0.003)	-0.002	(-0.008, 0.004)	-0.002	(-0.008, 0.003)
$\beta_{os,1}^{\pi}$	-1.072	(-1.344, -0.767)	-1.004	(-1.337, -0.718)	-1.076	(-1.368, -0.793)	-1.295	(-1.652, -0.976)	-0.807	(-1.051, -0.559)
$\beta_{os,2}^{\pi}$	-0.242	(-0.479, -0.009)	-0.183	(-0.432, 0.051)	-0.221	(-0.434, -0.001)	-0.382	(-0.667, -0.116)	-0.064	(-0.279, 0.149)
$\beta_{os,3}^{\pi}$	0.069	(-0.204, 0.34)	0.113	(-0.119, 0.388)	0.066	(-0.165, 0.294)	-0.067	(-0.326, 0.18)	0.217	(-0.01, 0.465)
$\beta_{pfs,1}^{\pi}$	-1.845	(-2.166, -1.564)	-1.834	(-2.156, -1.516)	-1.829	(-2.136, -1.519)	-1.822	(-2.134, -1.515)	-1.847	(-2.182, -1.523)
$\beta_{pfs,2}^{\pi}$	-0.623	(-0.857, -0.383)	-0.624	(-0.874, -0.372)	-0.619	(-0.848, -0.403)	-0.624	(-0.856, -0.39)	-0.612	(-0.864, -0.364)
$\beta_{pfs,3}^{\pi}$	-0.370	(-0.602, -0.125)	-0.362	(-0.583, -0.131)	-0.358	(-0.592, -0.124)	-0.347	(-0.58, -0.119)	-0.356	(-0.595, -0.132)
π_1^{os}	0.256	(0.207, 0.317)	0.269	(0.208, 0.328)	0.255	(0.203, 0.311)	0.216	(0.161, 0.274)	0.309	(0.259, 0.364)
π_2^{os}	0.440	(0.383, 0.498)	0.454	(0.394, 0.513)	0.445	(0.393, 0.5)	0.406	(0.339, 0.471)	0.484	(0.431, 0.537)
π_3^{os}	0.517	(0.449, 0.584)	0.528	(0.47, 0.596)	0.516	(0.459, 0.573)	0.483	(0.419, 0.545)	0.554	(0.497, 0.614)
π_1^{pfs}	0.138	(0.103, 0.173)	0.139	(0.104, 0.18)	0.139	(0.106, 0.18)	0.140	(0.106, 0.18)	0.137	(0.101, 0.179)
π_2^{pfs}	0.350	(0.298, 0.405)	0.349	(0.294, 0.408)	0.351	(0.3, 0.401)	0.349	(0.298, 0.404)	0.352	(0.297, 0.41)
π_3^{pfs}	0.409	(0.354, 0.469)	0.411	(0.358, 0.467)	0.412	(0.356, 0.469)	0.414	(0.359, 0.47)	0.412	(0.356, 0.467)

1OS distribution/PFS distribution.

Table 9 Posterior summary statistics for separate mixture cure models parameters and all OS distributions with log-logistic PFS distribution.

	**Exp/Log-logistic**[1]		**Weibull/Log-logistic**		**Gompertz/Log-logistic**		**Log-logistic/Log-logistic**		**log-Normal/Log-logistic**	
**Parameter**	**Mean**	**95% CrI**	**Mean**	**95% CrI**	**Mean**	**95% CrI**	**Mean**	**95% CrI**	**Mean**	**95% CrI**
β_0^{os}	-3.047	(-3.201, -2.914)	2.995	(2.875, 3.127)	-3.048	(-3.183, -2.91)	2.717	(2.578, 2.867)	2.477	(2.405, 2.543)
β_1^{os}	-0.012	(-0.021, -0.003)	0.009	(0.001, 0.018)	-0.012	(-0.02, -0.002)	0.008	(-0.001, 0.017)	0.000	(-0.004, 0.005)
β_0^{pfs}	1.328	(1.262, 1.395)	1.329	(1.26, 1.4)	1.331	(1.263, 1.406)	1.328	(1.253, 1.417)	1.326	(1.258, 1.401)
β_1^{pfs}	0.002	(-0.002, 0.007)	0.002	(-0.002, 0.007)	0.002	(-0.003, 0.007)	0.002	(-0.002, 0.007)	0.002	(-0.003, 0.007)
$\beta_{os,1}^{\pi}$	-1.087	(-1.409, -0.804)	-1.001	(-1.3, -0.704)	-1.061	(-1.364, -0.799)	-1.300	(-1.69, -0.952)	-0.821	(-1.055, -0.569)
$\beta_{os,2}^{\pi}$	-0.239	(-0.473, -0.007)	-0.200	(-0.423, 0.051)	-0.243	(-0.471, 0.006)	-0.383	(-0.661, -0.099)	-0.062	(-0.296, 0.162)
$\beta_{os,3}^{\pi}$	0.060	(-0.169, 0.294)	0.092	(-0.13, 0.329)	0.069	(-0.17, 0.318)	-0.073	(-0.305, 0.195)	0.214	(-0.018, 0.425)
$\beta_{pfs,1}^{\pi}$	-1.870	(-2.199, -1.517)	-1.866	(-2.228, -1.535)	-1.862	(-2.201, -1.539)	-1.871	(-2.207, -1.566)	-1.864	(-2.18, -1.549)
$\beta_{pfs,2}^{\pi}$	-0.618	(-0.875, -0.372)	-0.619	(-0.855, -0.372)	-0.621	(-0.858, -0.369)	-0.618	(-0.858, -0.356)	-0.610	(-0.838, -0.381)
$\beta_{pfs,3}^{\pi}$	-0.360	(-0.596, -0.148)	-0.347	(-0.569, -0.12)	-0.348	(-0.568, -0.083)	-0.358	(-0.581, -0.122)	-0.330	(-0.547, -0.121)
π_1^{os}	0.253	(0.196, 0.309)	0.270	(0.214, 0.331)	0.258	(0.204, 0.31)	0.216	(0.156, 0.279)	0.306	(0.258, 0.362)
π_2^{os}	0.441	(0.384, 0.498)	0.450	(0.396, 0.513)	0.440	(0.384, 0.502)	0.406	(0.341, 0.475)	0.485	(0.427, 0.541)
π_3^{os}	0.515	(0.458, 0.573)	0.523	(0.468, 0.581)	0.517	(0.458, 0.579)	0.482	(0.424, 0.549)	0.553	(0.495, 0.605)
π_1^{pfs}	0.135	(0.1, 0.18)	0.135	(0.097, 0.177)	0.136	(0.1, 0.177)	0.135	(0.099, 0.173)	0.135	(0.102, 0.175)
π_2^{pfs}	0.351	(0.294, 0.408)	0.351	(0.298, 0.408)	0.350	(0.298, 0.409)	0.351	(0.298, 0.412)	0.352	(0.302, 0.406)
π_3^{pfs}	0.411	(0.355, 0.463)	0.414	(0.361, 0.47)	0.414	(0.362, 0.479)	0.412	(0.359, 0.47)	0.418	(0.366, 0.47)

1OS distribution/PFS distribution.

Table 10 Posterior summary statistics for separate mixture cure models parameters and all OS distributions with log-Normal PFS distribution.

	**Exp/log-Normal**[1]		**Weibull/log-Normal**		**Gompertz/log-Normal**		**Log-logistic/log-Normal**		**log-Normal/log-Normal**	
**Parameter**	**Mean**	**95% CrI**	**Mean**	**95% CrI**	**Mean**	**95% CrI**	**Mean**	**95% CrI**	**Mean**	**95% CrI**
β_0^{os}	-3.046	(-3.183, -2.909)	2.996	(2.886, 3.113)	-3.039	(-3.186, -2.891)	2.714	(2.572, 2.868)	2.478	(2.41, 2.543)
β_1^{os}	-0.012	(-0.02, -0.003)	0.009	(0.001, 0.017)	-0.012	(-0.02, -0.002)	0.007	(-0.002, 0.016)	0.000	(-0.004, 0.005)
β_0^{pfs}	1.247	(1.198, 1.294)	1.248	(1.197, 1.29)	1.249	(1.202, 1.295)	1.248	(1.204, 1.293)	1.247	(1.204, 1.294)
β_1^{pfs}	0.000	(-0.004, 0.003)	0.000	(-0.003, 0.003)	0.000	(-0.003, 0.003)	0.000	(-0.003, 0.003)	0.000	(-0.003, 0.003)
$\beta_{os,1}^{\pi}$	-1.082	(-1.378, -0.824)	-1.002	(-1.346, -0.756)	-1.073	(-1.383, -0.775)	-1.292	(-1.71, -0.936)	-0.808	(-1.052, -0.61)
$\beta_{os,2}^{\pi}$	-0.244	(-0.487, 0.022)	-0.176	(-0.423, 0.086)	-0.224	(-0.462, 0.011)	-0.398	(-0.7, -0.097)	-0.061	(-0.305, 0.158)
$\beta_{os,3}^{\pi}$	0.056	(-0.178, 0.28)	0.110	(-0.107, 0.362)	0.062	(-0.192, 0.33)	-0.073	(-0.324, 0.193)	0.212	(-0.006, 0.443)
$\beta_{pfs,1}^{\pi}$	-1.575	(-1.904, -1.281)	-1.558	(-1.861, -1.298)	-1.565	(-1.889, -1.249)	-1.553	(-1.839, -1.268)	-1.565	(-1.891, -1.272)
$\beta_{pfs,2}^{\pi}$	-0.307	(-0.515, -0.048)	-0.305	(-0.553, -0.066)	-0.307	(-0.519, -0.098)	-0.301	(-0.54, -0.056)	-0.307	(-0.538, -0.088)
$\beta_{pfs,3}^{\pi}$	-0.067	(-0.321, 0.215)	-0.071	(-0.303, 0.189)	-0.060	(-0.309, 0.176)	-0.056	(-0.273, 0.164)	-0.056	(-0.322, 0.182)
π_1^{os}	0.254	(0.201, 0.305)	0.270	(0.207, 0.32)	0.256	(0.201, 0.315)	0.217	(0.153, 0.282)	0.309	(0.259, 0.352)
π_2^{os}	0.440	(0.381, 0.506)	0.456	(0.396, 0.521)	0.444	(0.387, 0.503)	0.402	(0.332, 0.476)	0.485	(0.424, 0.54)
π_3^{os}	0.514	(0.456, 0.57)	0.527	(0.473, 0.59)	0.515	(0.452, 0.582)	0.482	(0.42, 0.548)	0.553	(0.498, 0.609)
π_1^{pfs}	0.173	(0.13, 0.217)	0.175	(0.135, 0.215)	0.174	(0.131, 0.223)	0.176	(0.137, 0.22)	0.174	(0.131, 0.219)
π_2^{pfs}	0.424	(0.374, 0.488)	0.425	(0.365, 0.484)	0.424	(0.373, 0.476)	0.426	(0.368, 0.486)	0.424	(0.369, 0.478)
π_3^{pfs}	0.483	(0.42, 0.554)	0.482	(0.425, 0.547)	0.485	(0.423, 0.544)	0.486	(0.432, 0.541)	0.486	(0.42, 0.545)

1OS distribution/PFS distribution.

Table 11 WAIC point estimates (and standard errors) for hierarchical model and all distributions.

OS distn	PFS distn	ELPD†	$p_D^{\ddagger}$	WAIC
exp	exp	-5064.82 (80.38)	11.16 (0.46)	10129.63 (160.77)
	Gompertz	-5065.64 (80.44)	11.8 (0.49)	10131.29 (160.88)
	loglogistic	-4970.34 (80.72)	11.78 (0.33)	9940.68 (161.45)
	lognormal	-4730.47 (92.79)	18.04 (0.91)	9460.94 (185.59)
	Weibull	-5062.99 (81.69)	14.63 (0.8)	10125.97 (163.38)
Gompertz	exp	-5064.99 (80.41)	11.45 (0.45)	10129.99 (160.82)
	Gompertz	-5065.61 (80.44)	11.66 (0.48)	10131.22 (160.88)
	loglogistic	-4970.42 (80.79)	11.68 (0.34)	9940.84 (161.59)
	lognormal	-4730.92 (92.64)	18.31 (0.95)	9461.84 (185.29)
	Weibull	-5063.81 (81.74)	15.45 (0.85)	10127.61 (163.48)
loglogistic	exp	-5059.86 (80.47)	11.29 (0.42)	10119.73 (160.93)
	Gompertz	-5060.37 (80.4)	11.64 (0.45)	10120.74 (160.8)
	loglogistic	-4966.22 (80.75)	12.77 (0.35)	9932.44 (161.5)
	lognormal	-4726.57 (93.17)	19.43 (0.91)	9453.13 (186.34)
	Weibull	-5058.17 (81.84)	14.8 (0.74)	10116.33 (163.67)
lognormal	exp	-4972.55 (84.58)	16.35 (0.61)	9945.09 (169.15)
	Gompertz	-4973.16 (84.69)	16.5 (0.56)	9946.32 (169.38)
	loglogistic	-4877.87 (85.79)	16.53 (0.5)	9755.74 (171.59)
	lognormal	-4638.85 (100.27)	23.58 (1)	9277.71 (200.55)
	Weibull	-4970.22 (86.17)	19.01 (0.89)	9940.44 (172.34)
Weibull	exp	-5063.26 (80.7)	11.93 (0.46)	10126.53 (161.4)
	Gompertz	-5064.37 (80.64)	12.67 (0.52)	10128.73 (161.28)
	loglogistic	-4968.74 (81.03)	12.3 (0.37)	9937.49 (162.07)
	lognormal	-4729.78 (93.18)	19.54 (0.97)	9459.56 (186.36)
	Weibull	-5061.91 (82.05)	15.77 (0.85)	10123.81 (164.11)

†ELPD: Expected log pointwise predictive density; ‡p_D: Effective number of parameters; WAIC: widely applicable information criterion.

Table 17 shows the survival estimates at 60 months extrapolated using 12 and 30 months data cut point. Clearly, when the survival curve has converged to the long-term plateau this is equivalent to the cure fraction.

8 | POSTERIOR PREDICTIVE CHECKS

New event time data generated according to the following.

$$p(t^{\text{pred}} \mid \mathbf{T}, \mathbf{X}) = \int_{\Theta} p(t^{\text{pred}} \mid \theta, \mathbf{X}) p(\theta \mid \mathbf{t}, \mathbf{X}) \, \mathrm{d}\theta$$

9 | DAGS

Figures 17 and 18 show DAGs for the complete pooling and no pooling cure fraction models, respectively.

Table 12 WAIC point estimates (and standard errors) for separate models and all distributions.

**OS distn**	**PFS distn**	**ELPD**[†]	$p_D^{\ddagger}$	**WAIC**
exp	exp	-5066.38 (81.4)	10.46 (0.43)	10132.76 (162.81)
	Gompertz	-5066.68 (81.46)	10.38 (0.46)	10133.37 (162.92)
	loglogistic	-4971.73 (81.67)	10.36 (0.28)	9943.46 (163.34)
	lognormal	-4732.41 (93.18)	18.56 (0.99)	9464.83 (186.36)
	Weibull	-5064.87 (82.86)	14.39 (0.86)	10129.74 (165.72)
Gompertz	exp	-5066.72 (81.44)	10.51 (0.45)	10133.43 (162.89)
	Gompertz	-5066.76 (81.49)	10.19 (0.44)	10133.53 (162.97)
	loglogistic	-4972.24 (81.7)	10.7 (0.28)	9944.47 (163.4)
	lognormal	-4732.23 (93.3)	18.01 (0.95)	9464.46 (186.61)
	Weibull	-5064.46 (82.92)	13.5 (0.78)	10128.91 (165.83)
loglogistic	exp	-5062.51 (81.89)	10.79 (0.4)	10125.02 (163.78)
	Gompertz	-5062.64 (82.01)	10.26 (0.41)	10125.27 (164.02)
	loglogistic	-4968.45 (82.36)	11.63 (0.28)	9936.89 (164.72)
	lognormal	-4728.25 (94.2)	18.18 (0.92)	9456.51 (188.4)
	Weibull	-5060.18 (83.31)	13.79 (0.69)	10120.37 (166.62)
lognormal	exp	-4973.43 (85.26)	14.63 (0.55)	9946.86 (170.53)
	Gompertz	-4973.48 (85.22)	14.31 (0.51)	9946.96 (170.44)
	loglogistic	-4879.07 (86.55)	15.07 (0.44)	9758.14 (173.09)
	lognormal	-4638.43 (100.4)	21.3 (0.96)	9276.85 (200.8)
	Weibull	-4972.48 (87.07)	19.54 (0.91)	9944.95 (174.13)
Weibull	exp	-5064.74 (81.52)	11.11 (0.45)	10129.48 (163.04)
	Gompertz	-5065.16 (81.59)	11.16 (0.44)	10130.32 (163.18)
	loglogistic	-4970.64 (81.87)	11.68 (0.35)	9941.27 (163.73)
	lognormal	-4730.93 (93.78)	19.04 (0.96)	9461.85 (187.56)
	Weibull	-5063.2 (83.09)	14.87 (0.83)	10126.41 (166.17)

[†]ELPD: Expected log pointwise predictive density; $^{\ddagger}p_D$: Effective number of parameters; WAIC: widely applicable information criterion.

Table 13 WAIC statistics for all distributions and hierarchical mixture cure model.

		ELPD[†]		$p_D^{\ddagger}$		WAIC	
**OS**	**PFS**	**Estimate**	**SE**	**Estimate**	**SE**	**Estimate**	**SE**
exp	exp	-5064.70	80.37	11.13	0.46	10129.40	160.74
	gompertz	-5065.64	80.44	11.80	0.49	10131.29	160.88
	loglogistic	-4970.34	80.72	11.78	0.33	9940.68	161.45
	lognormal	-4730.47	92.79	18.04	0.91	9460.94	185.59
	weibull	-5062.99	81.69	14.63	0.80	10125.97	163.38
gompertz	exp	-5064.99	80.41	11.45	0.45	10129.99	160.82
	gompertz	-5065.61	80.44	11.66	0.48	10131.22	160.88
	loglogistic	-4970.42	80.79	11.68	0.34	9940.84	161.59
	lognormal	-4730.92	92.64	18.31	0.95	9461.84	185.29
	weibull	-5063.81	81.74	15.45	0.85	10127.61	163.48
loglogistic	exp	-5059.86	80.47	11.29	0.42	10119.73	160.93
	gompertz	-5060.37	80.40	11.64	0.45	10120.74	160.80
	loglogistic	-4966.22	80.75	12.77	0.35	9932.44	161.50
	lognormal	-4726.57	93.17	19.43	0.91	9453.13	186.34
	weibull	-5058.17	81.84	14.80	0.74	10116.33	163.67
lognormal	exp	-4972.55	84.58	16.35	0.61	9945.09	169.15
	gompertz	-4973.16	84.69	16.50	0.56	9946.32	169.38
	loglogistic	-4877.87	85.79	16.53	0.50	9755.74	171.59
	lognormal	-4638.85	100.27	23.58	1.00	9277.71	200.55
	weibull	-4970.22	86.17	19.01	0.89	9940.44	172.34
weibull	exp	-5063.26	80.70	11.93	0.46	10126.53	161.40
	gompertz	-5064.37	80.64	12.67	0.52	10128.73	161.28
	loglogistic	-4968.74	81.03	12.30	0.37	9937.49	162.07
	lognormal	-4729.78	93.18	19.54	0.97	9459.56	186.36
	weibull	-5061.91	82.05	15.77	0.85	10123.81	164.11

[†]ELPD: Expected log pointwise predictive density; $^{\ddagger}p_D$: Effective number of parameters

Table 14 WAIC statistics for all distributions and separate mixture cure models.

		ELPD[†]		$p_D^{\ddagger}$		WAIC	
**OS**	**PFS**	**Estimate**	**SE**	**Estimate**	**SE**	**Estimate**	**SE**
exp	exp	-5066.38	81.40	10.46	0.43	10132.76	162.81
	gompertz	-5066.68	81.46	10.38	0.46	10133.37	162.92
	loglogistic	-4971.73	81.67	10.36	0.28	9943.46	163.34
	lognormal	-4732.41	93.18	18.56	0.99	9464.83	186.36
	weibull	-5064.87	82.86	14.39	0.86	10129.74	165.72
gompertz	exp	-5066.72	81.44	10.51	0.45	10133.43	162.89
	gompertz	-5066.76	81.49	10.19	0.44	10133.53	162.97
	loglogistic	-4972.24	81.70	10.70	0.28	9944.47	163.40
	lognormal	-4732.23	93.30	18.01	0.95	9464.46	186.61
	weibull	-5064.46	82.92	13.50	0.78	10128.91	165.83
loglogistic	exp	-5062.51	81.89	10.79	0.40	10125.02	163.78
	gompertz	-5062.64	82.01	10.26	0.41	10125.27	164.02
	loglogistic	-4968.45	82.36	11.63	0.28	9936.89	164.72
	lognormal	-4728.25	94.20	18.18	0.92	9456.51	188.40
	weibull	-5060.18	83.31	13.79	0.69	10120.37	166.62
lognormal	exp	-4973.43	85.26	14.63	0.55	9946.86	170.53
	gompertz	-4973.48	85.22	14.31	0.51	9946.96	170.44
	loglogistic	-4879.07	86.55	15.07	0.44	9758.14	173.09
	lognormal	-4638.43	100.40	21.30	0.96	9276.85	200.80
	weibull	-4972.48	87.07	19.54	0.91	9944.95	174.13
weibull	exp	-5064.74	81.52	11.11	0.45	10129.48	163.04
	gompertz	-5065.16	81.59	11.16	0.44	10130.32	163.18
	loglogistic	-4970.64	81.87	11.68	0.35	9941.27	163.73
	lognormal	-4730.93	93.78	19.04	0.96	9461.85	187.56
	weibull	-5063.20	83.09	14.87	0.83	10126.41	166.17

[†]ELPD: Expected log pointwise predictive density; [‡]p_D: Effective number of parameters

Table 15 PSIS-LOO statistics for all distributions and hierarchical mixture cure model.

		ELPD[†]		$p_D^{\ddagger}$		LOO AIC	
**OS**	**PFS**	**Estimate**	**SE**	**Estimate**	**SE**	**Estimate**	**SE**
exp	exp	-5064.80	80.37	11.22	0.46	10129.59	160.75
	gompertz	-5065.74	80.44	11.90	0.49	10131.48	160.88
	loglogistic	-4970.43	80.73	11.87	0.33	9940.86	161.45
	lognormal	-4730.60	92.80	18.18	0.92	9461.20	185.59
	weibull	-5063.08	81.69	14.72	0.80	10126.17	163.38
gompertz	exp	-5065.09	80.41	11.55	0.45	10130.18	160.82
	gompertz	-5065.70	80.44	11.75	0.48	10131.40	160.88
	loglogistic	-4970.50	80.79	11.76	0.34	9940.99	161.59
	lognormal	-4731.06	92.65	18.45	0.96	9462.12	185.30
	weibull	-5063.92	81.74	15.57	0.86	10127.84	163.49
loglogistic	exp	-5059.95	80.47	11.38	0.42	10119.90	160.94
	gompertz	-5060.45	80.40	11.73	0.45	10120.90	160.80
	loglogistic	-4966.31	80.75	12.87	0.35	9932.63	161.50
	lognormal	-4726.71	93.18	19.58	0.92	9453.42	186.35
	weibull	-5058.26	81.84	14.90	0.74	10116.53	163.67
lognormal	exp	-4972.68	84.58	16.49	0.61	9945.37	169.16
	gompertz	-4973.28	84.69	16.62	0.57	9946.56	169.39
	loglogistic	-4878.00	85.79	16.67	0.50	9756.01	171.59
	lognormal	-4639.02	100.27	23.75	1.00	9278.04	200.55
	weibull	-4970.34	86.17	19.13	0.89	9940.68	172.34
weibull	exp	-5063.35	80.70	12.02	0.47	10126.69	161.40
	gompertz	-5064.46	80.64	12.76	0.53	10128.92	161.28
	loglogistic	-4968.83	81.03	12.39	0.37	9937.67	162.07
	lognormal	-4729.90	93.18	19.66	0.98	9459.80	186.36
	weibull	-5062.01	82.05	15.88	0.85	10124.03	164.11

[†]ELPD: Expected log pointwise predictive density; [‡]p_D: Effective number of parameters

Table 16 PSIS-LOO statistics for all distributions and separate mixture cure models.

		ELPD[†]		$p_D^{\ddagger}$		LOO AIC	
**OS**	**PFS**	**Estimate**	**SE**	**Estimate**	**SE**	**Estimate**	**SE**
exp	exp	-5066.47	81.40	10.55	0.43	10132.93	162.81
	gompertz	-5066.76	81.46	10.46	0.46	10133.52	162.92
	loglogistic	-4971.81	81.67	10.44	0.28	9943.61	163.34
	lognormal	-4732.55	93.18	18.69	0.99	9465.09	186.37
	weibull	-5064.95	82.86	14.48	0.86	10129.91	165.72
gompertz	exp	-5066.80	81.44	10.59	0.46	10133.60	162.89
	gompertz	-5066.84	81.49	10.27	0.44	10133.69	162.97
	loglogistic	-4972.33	81.70	10.79	0.29	9944.66	163.40
	lognormal	-4732.34	93.31	18.13	0.95	9464.68	186.61
	weibull	-5064.54	82.92	13.58	0.79	10129.08	165.84
loglogistic	exp	-5062.60	81.89	10.88	0.40	10125.20	163.78
	gompertz	-5062.72	82.01	10.34	0.41	10125.43	164.03
	loglogistic	-4968.53	82.36	11.72	0.29	9937.06	164.72
	lognormal	-4728.37	94.20	18.30	0.93	9456.75	188.40
	weibull	-5060.29	83.31	13.90	0.69	10120.59	166.62
lognormal	exp	-4973.54	85.26	14.74	0.56	9947.08	170.53
	gompertz	-4973.58	85.22	14.40	0.52	9947.15	170.44
	loglogistic	-4879.18	86.55	15.17	0.45	9758.35	173.10
	lognormal	-4638.57	100.40	21.44	0.96	9277.15	200.80
	weibull	-4972.61	87.07	19.67	0.92	9945.22	174.14
weibull	exp	-5064.82	81.52	11.18	0.45	10129.63	163.04
	gompertz	-5065.24	81.59	11.24	0.44	10130.48	163.18
	loglogistic	-4970.70	81.87	11.74	0.35	9941.40	163.73
	lognormal	-4731.06	93.78	19.18	0.97	9462.12	187.57
	weibull	-5063.28	83.09	14.95	0.82	10126.56	166.18

[†]ELPD: Expected log pointwise predictive density; [‡]p_D: Effective number of parameters

Table 17 Survival probability estimates for separate and hierarchical models at 60 months using 12, 30 and 60 months data cut points. Models with exponential or log-normal distributions for both OS and PFS are given.

Endpoint	Model	Distribution	Cut-point (month)	Ipilimumab	Nivolumab	Combined
OS	hier	exp	12	0.19 [0.14, 0.28]	0.34 [0.28, 0.39]	0.42 [0.33, 0.48]
OS	hier	exp	30	0.2 [0.11, 0.28]	0.33 [0.28, 0.38]	0.47 [0.4, 0.55]
OS	hier	exp	60	0.25 [0.2, 0.29]	0.31 [0.26, 0.36]	0.51 [0.46, 0.56]
OS	hier	lognormal	12	0.28 [0.23, 0.33]	0.38 [0.33, 0.43]	0.49 [0.43, 0.54]
OS	hier	lognormal	30	0.23 [0.17, 0.3]	0.33 [0.28, 0.38]	0.49 [0.43, 0.56]
OS	hier	lognormal	60	0.25 [0.21, 0.3]	0.31 [0.26, 0.35]	0.51 [0.46, 0.56]
OS	separate	exp	12	0.26 [0.18, 0.34]	0.34 [0.28, 0.4]	0.34 [0.23, 0.44]
OS	separate	exp	30	0.22 [0.17, 0.28]	0.32 [0.27, 0.38]	0.47 [0.4, 0.52]
OS	separate	exp	60	0.25 [0.21, 0.3]	0.3 [0.25, 0.35]	0.5 [0.45, 0.55]
OS	separate	lognormal	12	0.34 [0.26, 0.42]	0.38 [0.32, 0.43]	0.41 [0.33, 0.51]
OS	separate	lognormal	30	0.27 [0.22, 0.32]	0.33 [0.27, 0.37]	0.48 [0.42, 0.54]
OS	separate	lognormal	60	0.27 [0.22, 0.32]	0.3 [0.25, 0.35]	0.51 [0.45, 0.56]
PFS	hier	exp	12	0.1 [0.05, 0.15]	0.39 [0.34, 0.44]	0.38 [0.32, 0.44]
PFS	hier	exp	30	0.1 [0.07, 0.14]	0.41 [0.34, 0.49]	0.39 [0.33, 0.45]
PFS	hier	exp	60	0.08 [0.05, 0.12]	0.42 [0.37, 0.48]	0.38 [0.32, 0.44]
PFS	hier	lognormal	12	0.16 [0.11, 0.2]	0.47 [0.41, 0.51]	0.43 [0.38, 0.48]
PFS	hier	lognormal	30	0.1 [0.07, 0.14]	0.43 [0.38, 0.49]	0.4 [0.34, 0.45]
PFS	hier	lognormal	60	0.08 [0.05, 0.12]	0.43 [0.38, 0.48]	0.38 [0.32, 0.43]
PFS	separate	exp	12	0.1 [0.06, 0.15]	0.35 [0.25, 0.45]	0.4 [0.33, 0.46]
PFS	separate	exp	30	0.11 [0.08, 0.15]	0.41 [0.34, 0.48]	0.38 [0.32, 0.43]
PFS	separate	exp	60	0.1 [0.07, 0.14]	0.42 [0.37, 0.48]	0.37 [0.31, 0.42]
PFS	separate	lognormal	12	0.15 [0.11, 0.19]	0.43 [0.35, 0.52]	0.44 [0.39, 0.49]
PFS	separate	lognormal	30	0.12 [0.08, 0.15]	0.43 [0.36, 0.5]	0.39 [0.34, 0.45]
PFS	separate	lognormal	60	0.1 [0.07, 0.13]	0.43 [0.38, 0.48]	0.37 [0.31, 0.42]

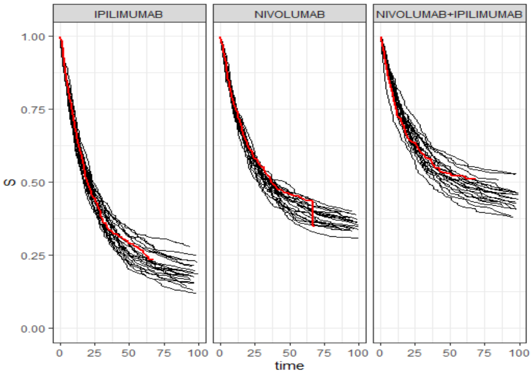

(**a**) OS data

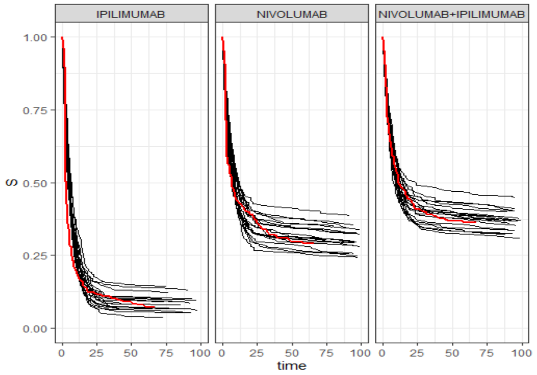

(**b**) PFS data

Figure 14 Posterior predictive values Kaplan-Meier curves for exponential OS and exponential PFS distributions with the hierarchical mixture cure model. Black lines represent end-points for 20 predicted cohorts and the red line is for the observed data.

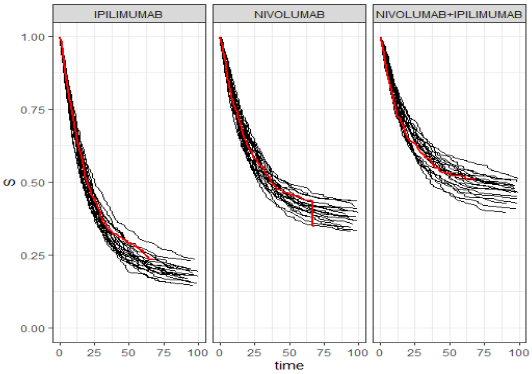

(**a**) OS data

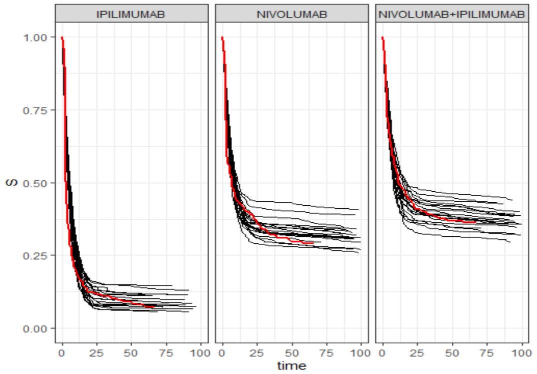

(**b**) PFS data

Figure 15 Posterior predictive values Kaplan-Meier curves for weibull OS and weibull PFS distributions with the hierarchical mixture cure model. Black lines represent end-points for 20 predicted cohorts and the red line is for the observed data.

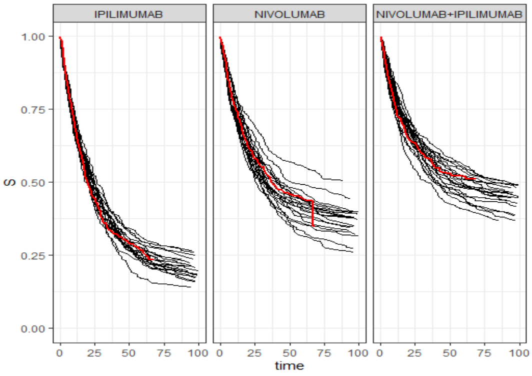

(**a**) OS data

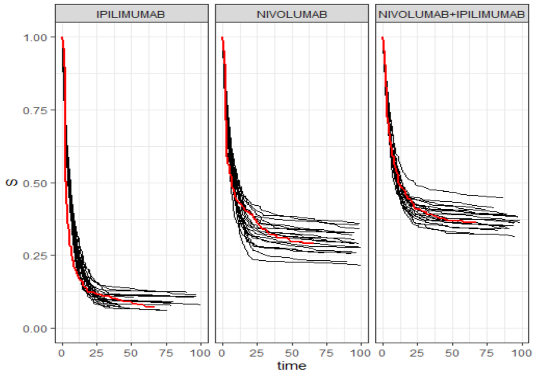

(**b**) PFS data

Figure 16 Posterior predictive values Kaplan-Meier curves for gompertz OS and gompertz PFS distributions with the hierarchical mixture cure model. Black lines represent end-points for 20 predicted cohorts and the red line is for the observed data.

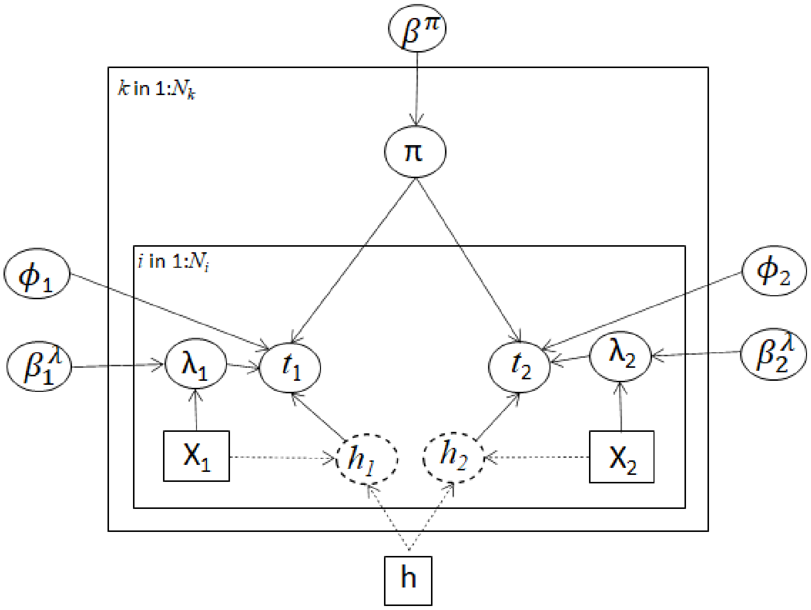

Figure 17 A complete cure fraction pooling mixture cure model DAG for a trial with two end-points. The trial indexes correspond to the motivating example end-points of PFS (1) and OS (2). Solid lines represent stochastic and dashed lines deterministic relationships, respectively. Cured patients have fixed hazards e.g. taken from life tables. The distribution of times for uncured patient regresses on covariates for the rate parameter and ϕ is the set of ancillary parameters.

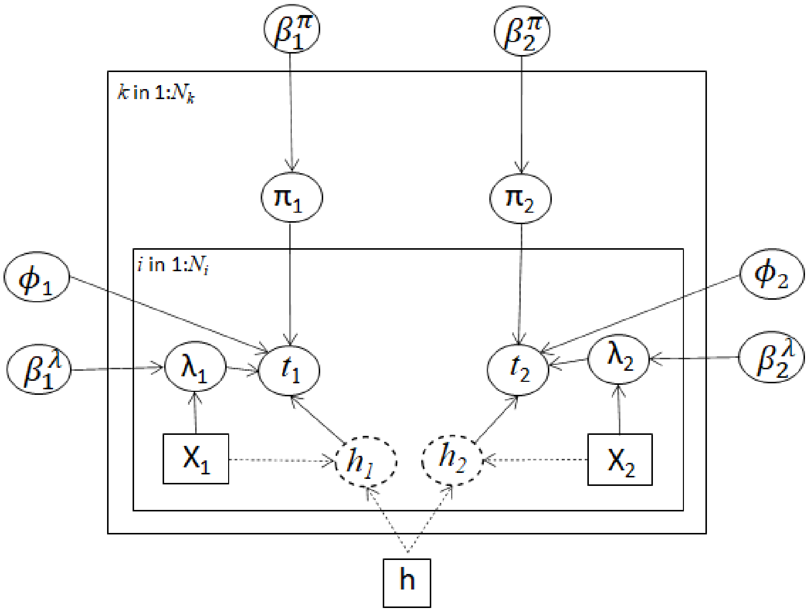

Figure 18 A no pooling cure fraction mixture cure model DAG for a trial with two end-points. The trial indexes correspond to the motivating example end-points of PFS (1) and OS (2). Solid lines represent stochastic and dashed lines deterministic relationships, respectively. Cured patients have fixed hazards e.g. taken from life tables. The distribution of times for uncured patient regresses on covariates for the rate parameter and ϕ is the set of ancillary parameters.

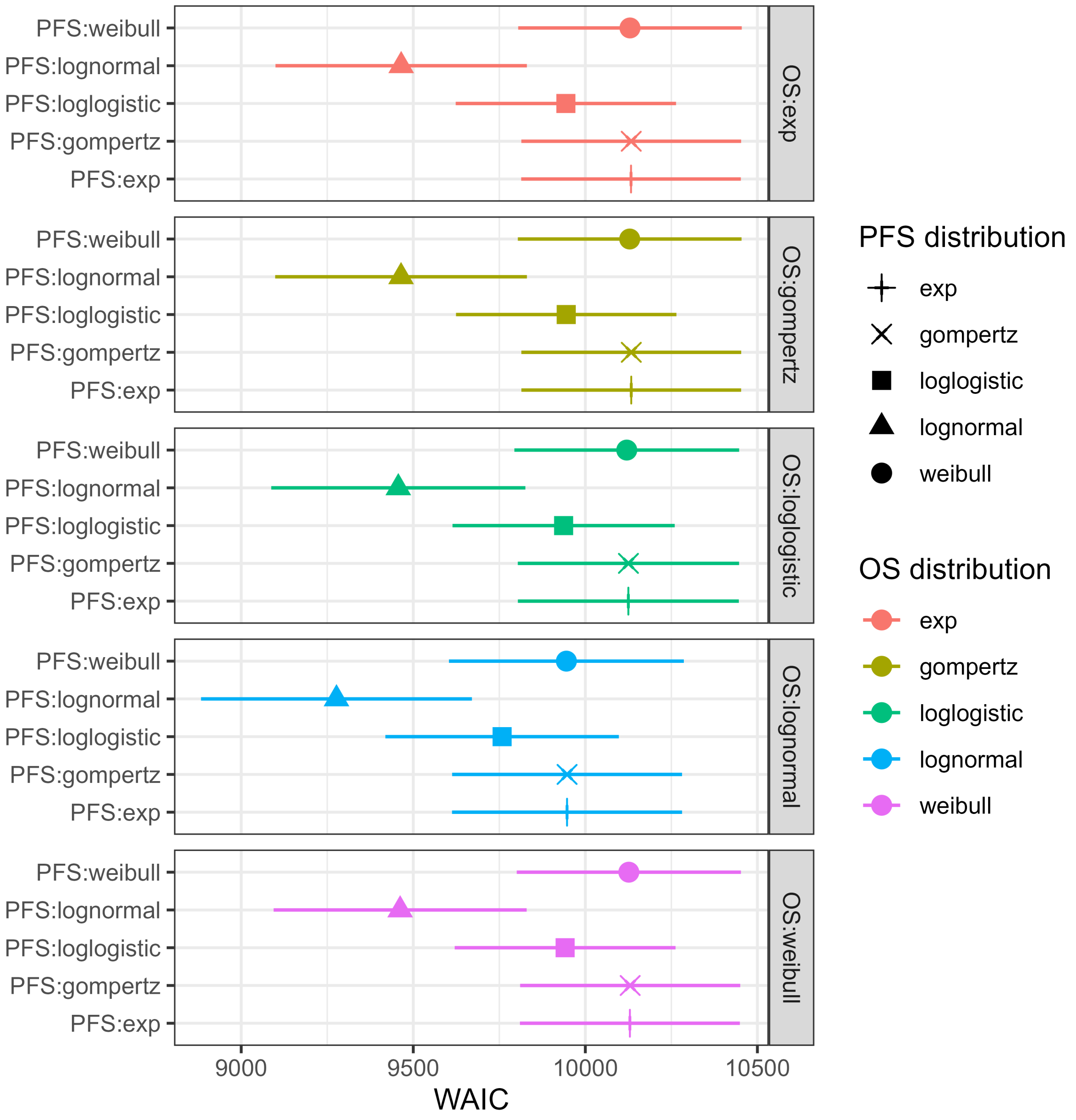

Figure 19 Separate model WAIC statistics.

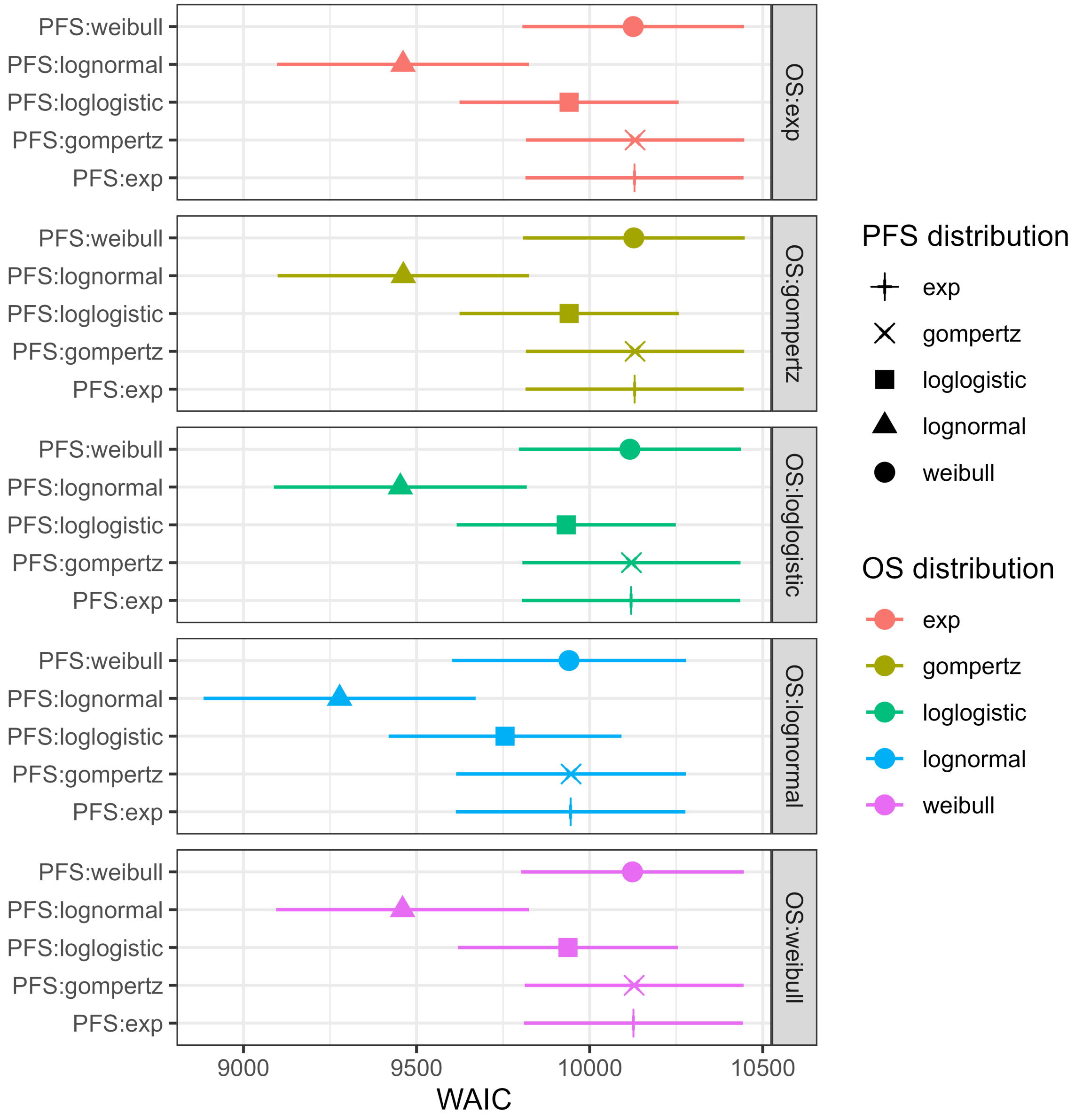

Figure 20 Hierarchical model WAIC statistics.

10 | SIMULATION STUDY

In this section, we present all of the plots for restricted mean survival time (RMST) and cure fraction (π) target estimands, and for the performance measures of bias and coverage.

Table 18 shows the simulation study scenarios input values. All scenarios are used for both the hierarchical model and separate model analyses. See the main text for the definitions of weak and informative for each parameter.

Scenario	n_e	n_{sim}	θ^u	σ	π
1	3	10	informative	informative	informative
2	10	10	informative	informative	informative
3	3	100	informative	informative	informative
4	10	100	informative	informative	informative
5	3	10	weak	informative	informative
6	10	10	weak	informative	informative
7	3	100	weak	informative	informative
8	10	100	weak	informative	informative
9	3	10	informative	weak	informative
10	10	10	informative	weak	informative
11	3	100	informative	weak	informative
12	10	100	informative	weak	informative
13	3	10	weak	weak	informative
14	10	10	weak	weak	informative
15	3	100	weak	weak	informative
16	10	100	weak	weak	informative
17	3	10	informative	informative	weak
18	10	10	informative	informative	weak
19	3	100	informative	informative	weak
20	10	100	informative	informative	weak
21	3	10	weak	informative	weak
22	10	10	weak	informative	weak
23	3	100	weak	informative	weak
24	10	100	weak	informative	weak
25	3	10	informative	weak	weak
26	10	10	informative	weak	weak
27	3	100	informative	weak	weak
28	10	100	informative	weak	weak
29	3	10	weak	weak	weak
30	10	10	weak	weak	weak
31	3	100	weak	weak	weak
32	10	100	weak	weak	weak

Table 18 Simulation study scenarios input values. All scenarios are used for both the hierarchical model and separate model analyses. n_e are the number of survival curves/groups, n_{sim} are the sample sizes, θ^u are the uncured survival model parameters, σ are the between-group standard deviations, and π are the global cure fraction means.

As a model check, we simulated data in a deterministic analysis. Scenarios were selected so that the cure fractions were not randomly sampled but taken from equally spaced quantiles of the underlying global cure fraction distribution, e.g. defining $Q^{\pi}(p)$ as the p-th quantile for the global cure fraction distribution on the logit scale then, e.g. for the 3 end-points case $\text{logit}(s_{ij}) = Q^{\pi}(p_j)$, where $p_j = 0.25, 0.5, 0.75$. In this way, we ensured that larger variances σ^2_{true} corresponded to wider spread of cure fractions. Also, individuals were strictly split in to a specified proportion of censored or non-censored exactly, rather than via e.g. Binomial sampling, in order to avoid introducing additional unnecessary noise. That is, we fixed the proportion of cured individuals so that $\sum_i c_{ij}/n_{\text{sim}} = p^{\text{cens}}$. The survival plots corresponding to these simulated input data are shown in Figure 21.

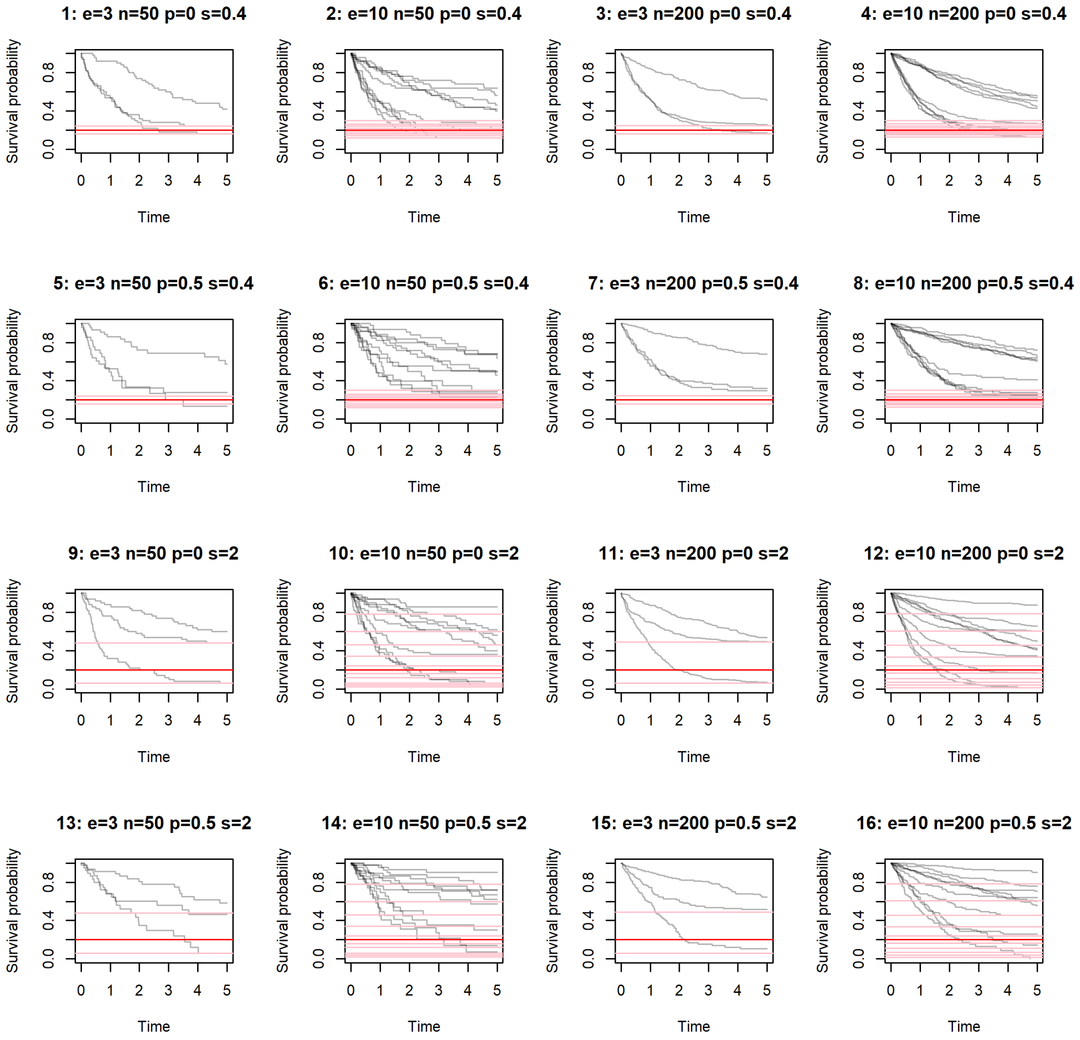

Figure 21 The survival plots for the simulated input data scenarios with deterministic cure fractions. The horizontal red lines indicate the cure fractions corresponding to each curve.

Heat map versions of the summary output table given in Table 1 in the main paper are presented in Figure 22 and Figure 23. The histograms of $\hat{\pi}$, $\widehat{RMST}$ are given in Figure 24 and Figure 25.

This section gives plots comparing the hierarchical and separate model results for all of the simulated data scenarios. Figures 26, 27 show lollipop plots for the coverage. Figures 28, 29 show lollipop plots for the bias. Figures 30, 31 show lollipop plots for the relative bias.

Scenario	Bias.hier.RMST	Bias.sep.RMST	EmpSE.hier.RMST	EmpSE.sep.RMST	RB.hier.RMST	RB.sep.RMST	Bias.hier.cf	Bias.sep.cf	EmpSE.hier.cf	EmpSE.sep.cf	RB.hier.cf	RB.sep.cf
1	0.01	0.01	0.03	0	0	0	0	0	0.01	0	0.01	0
2	0.01	0.01	0.03	0	0	0	0	0	0.01	0	0.01	0
3	0.01	0.01	0.03	0.01	0	0	0	0	0.01	0	0	0
4	0.01	0.01	0.03	0.01	0	0	0	0	0.01	0	0	0
5	0.57	0.5	0.22	0.16	0.18	0.16	0.04	0	0.08	0	0.15	0.01
6	0.55	0.42	0.28	0.21	0.19	0.14	0.06	0	0.09	0	0.22	0.01
7	0.57	0.39	0.14	0.09	0.19	0.14	0.12	0.01	0.05	0.01	0.46	0.05
8	0.52	0.37	0.15	0.1	0.18	0.14	0.1	0.01	0.05	0	0.36	0.04
9	0.01	0.01	0.06	0.01	0	0	0	0	0.02	0	0.01	0
10	0.01	0.01	0.06	0.01	0.01	0	0	0	0.02	0	0.01	0.01
11	0.01	0.01	0.04	0.02	0	0	0	0	0.02	0.01	0	0
12	0.01	0.01	0.04	0.03	0	0	0	0	0.01	0.01	0	0
13	0.61	0.51	0.23	0.16	0.2	0.16	0.06	0	0.09	0	0.21	0.02
14	0.57	0.43	0.27	0.22	0.2	0.14	0.07	0	0.09	0	0.27	0.02
15	0.57	0.42	0.14	0.09	0.19	0.14	0.12	0.03	0.05	0.01	0.47	0.11
16	0.51	0.39	0.15	0.1	0.18	0.15	0.1	0.02	0.05	0.01	0.36	0.09
17	0.2	0.36	0.19	0.2	0.08	0.13	0.07	0.13	0.07	0.07	0.26	0.5
18	0.08	0.38	0.15	0.23	0.03	0.16	0.02	0.13	0.05	0.07	0.09	0.48
19	0.03	0.08	0.1	0.14	0.01	0.03	0.01	0.03	0.04	0.05	0.03	0.1
20	0.01	0.08	0.06	0.15	0.01	0.03	0	0.03	0.02	0.05	0.01	0.1
21	0.91	0.9	0.19	0.21	0.32	0.31	0.2	0.21	0.08	0.08	0.75	0.78
22	0.84	0.87	0.21	0.24	0.32	0.33	0.17	0.2	0.05	0.09	0.65	0.74
23	0.63	0.62	0.15	0.14	0.22	0.21	0.14	0.15	0.05	0.05	0.53	0.56
24	0.56	0.57	0.15	0.15	0.21	0.21	0.11	0.12	0.05	0.05	0.41	0.45
25	0.19	0.36	0.2	0.21	0.07	0.13	0.07	0.13	0.07	0.07	0.25	0.5
26	0.08	0.38	0.15	0.23	0.03	0.15	0.02	0.13	0.05	0.07	0.09	0.48
27	0.03	0.08	0.1	0.14	0.01	0.03	0.01	0.03	0.04	0.05	0.03	0.1
28	0.01	0.08	0.06	0.15	0	0.03	0	0.03	0.02	0.05	0.01	0.1
29	0.91	0.91	0.2	0.2	0.32	0.32	0.2	0.21	0.08	0.09	0.74	0.78
30	0.84	0.87	0.22	0.25	0.32	0.33	0.17	0.2	0.06	0.09	0.65	0.73
31	0.63	0.62	0.14	0.14	0.22	0.21	0.14	0.15	0.05	0.05	0.54	0.56
32	0.56	0.57	0.15	0.15	0.2	0.21	0.11	0.12	0.05	0.05	0.41	0.45

Figure 22 Heat map of bias performance measures estimates for the hierarchical and separate models giving restricted mean survival time (RMST) and cure fraction (cf) results. Values are averaged over all curves within scenarios.

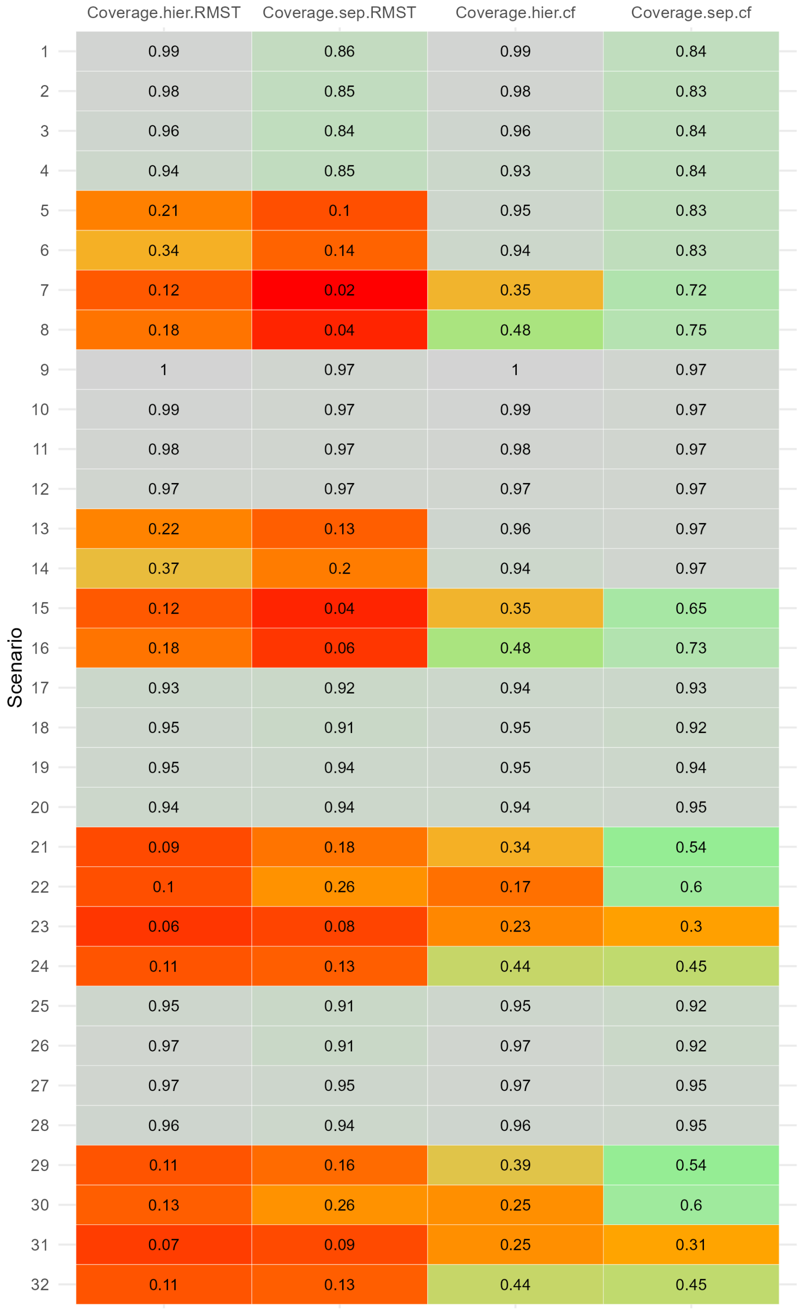

Figure 23 Heat map of coverage performance measure estimates for the hierarchical and separate models giving restricted mean survival time (RMST) and cure fraction (cf) results. Values are averaged over all curves within scenarios.

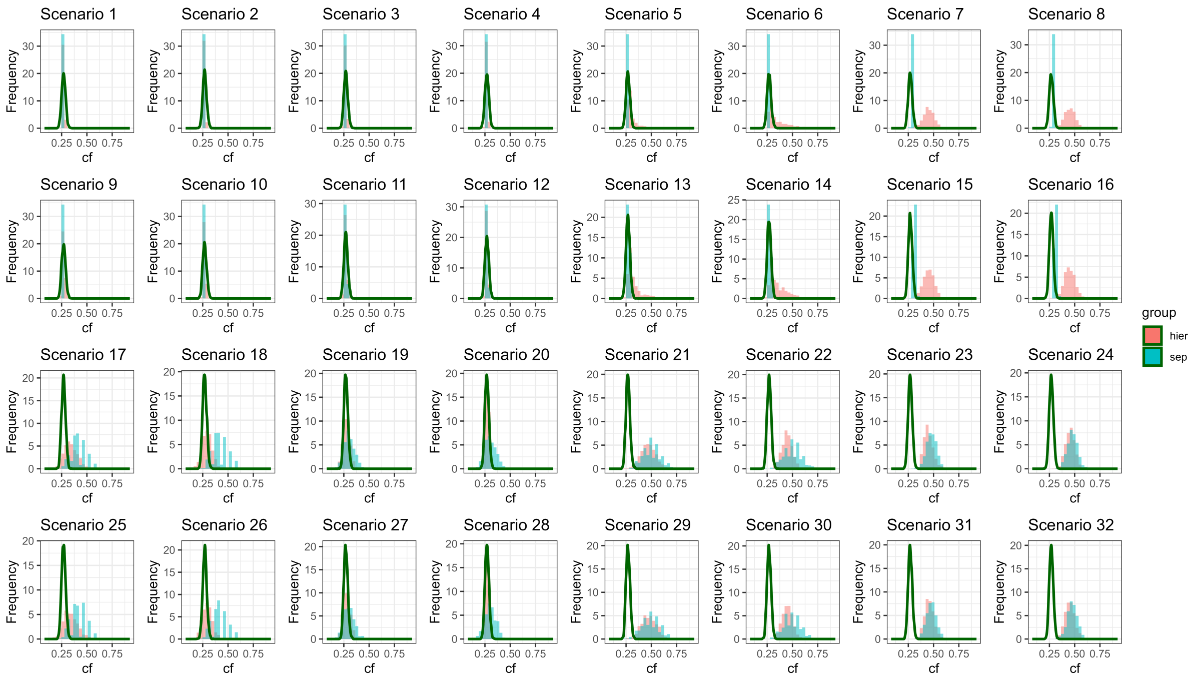

Figure 24 Histograms of posterior distributions of the estimated cure fraction ($\hat{\pi}$) for each of the simulation study scenarios.

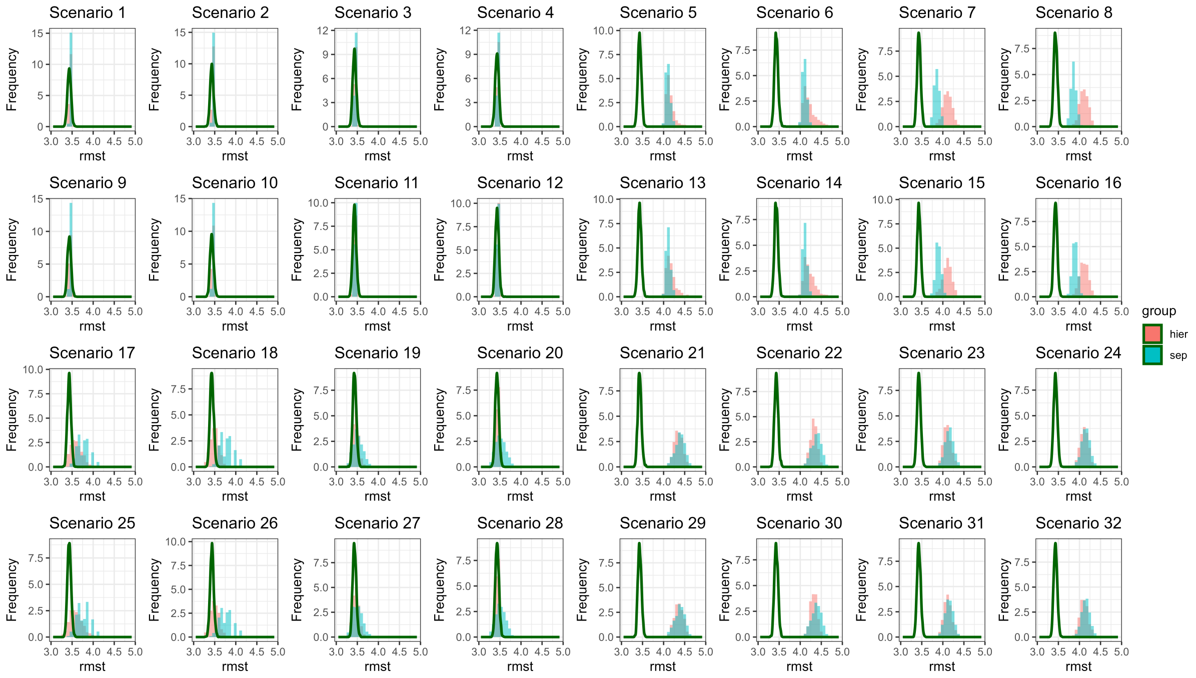

Figure 25 Histograms of posterior distributions of the estimated RMST ($\widehat{RMST}$) for each of the simulation study scenarios.

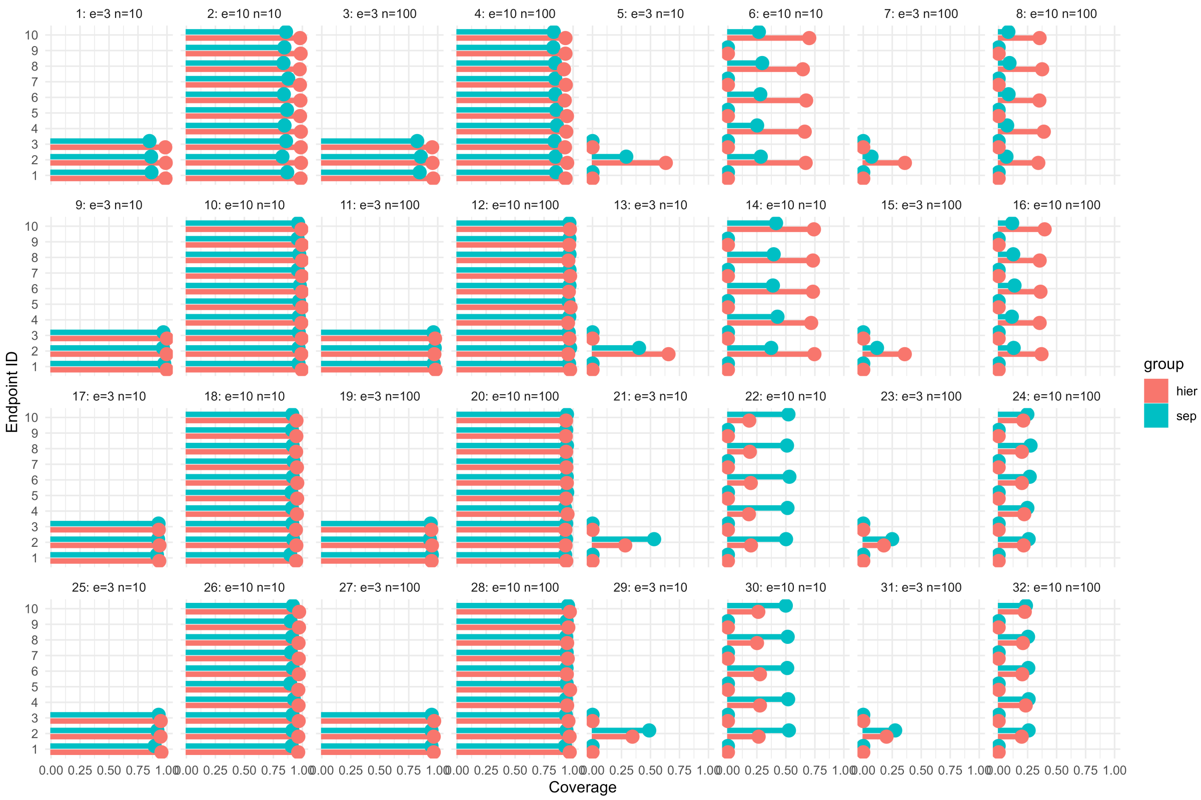

Figure 26 Lollipop plots of RMST coverage estimates for the different simulated data scenarios. The separate model is coloured blue and the hierarchical model is coloured red.

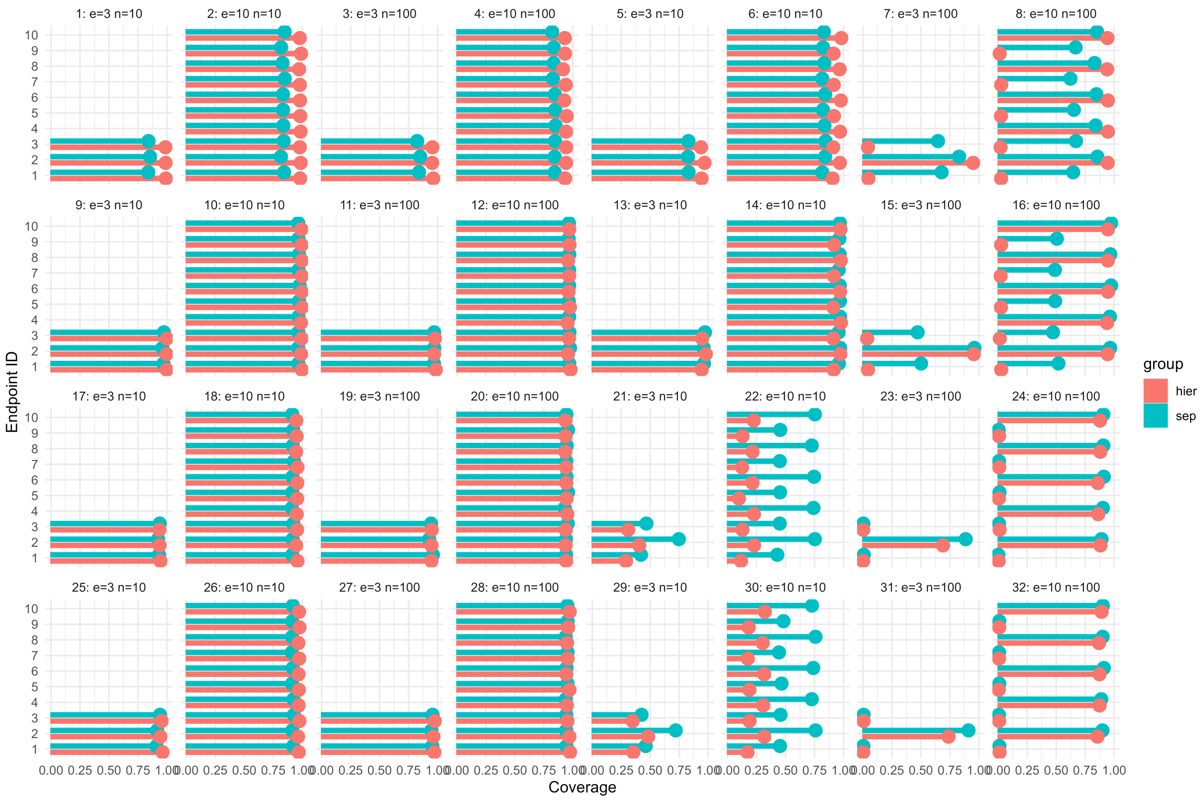

Figure 27 Lollipop plots of cure fraction coverage estimates for the different simulated data scenarios. The separate model is coloured blue and the hierarchical model is coloured red.

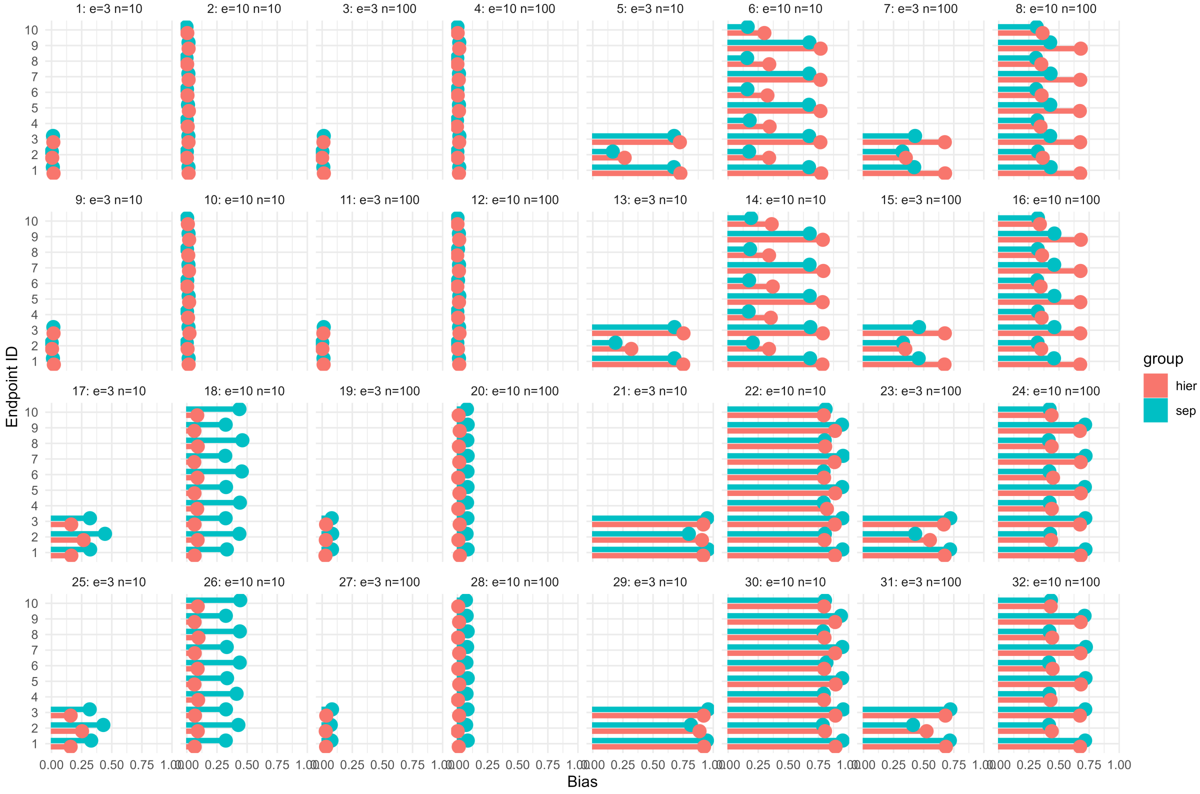

Figure 28 Lollipop plots of RMST bias estimates for the different simulated data deterministic scenarios. The separate model is coloured blue and the hierarchical model is coloured red.

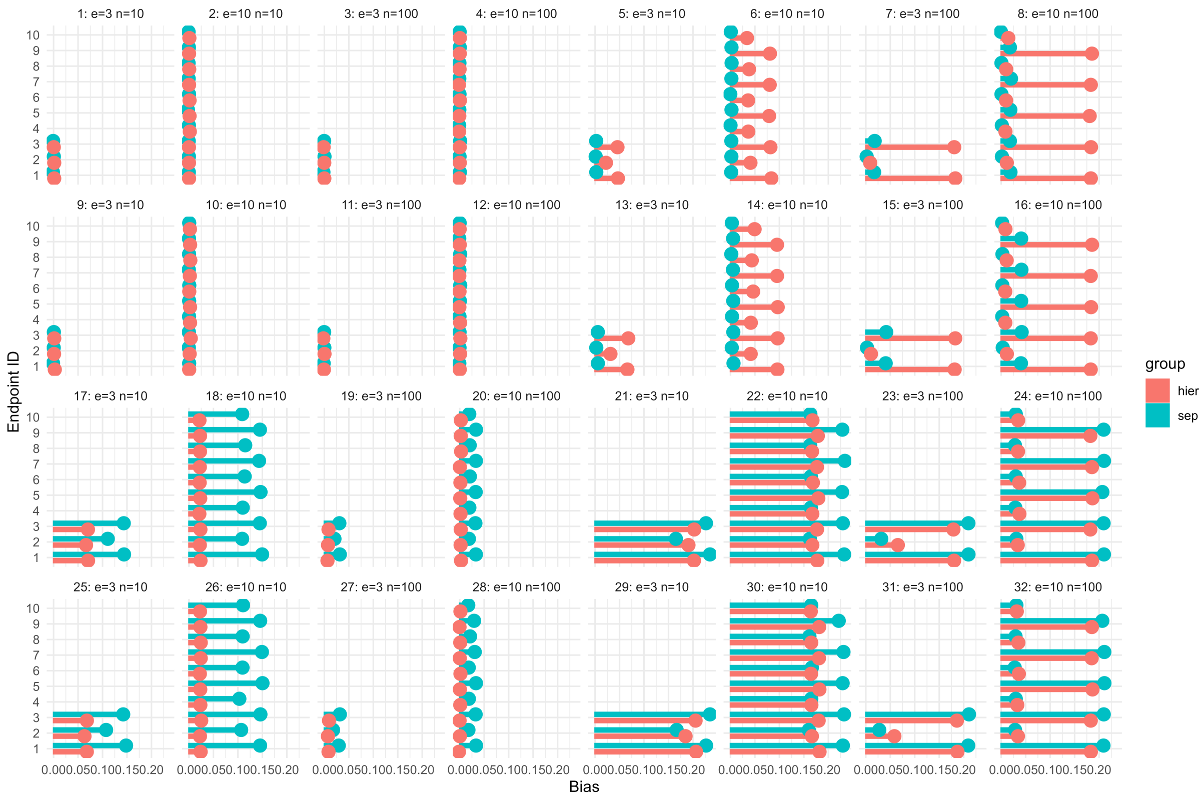

Figure 29 Lollipop plots of cure fraction bias estimates for the different simulated data scenarios. The separate model is coloured blue and the hierarchical model is coloured red.

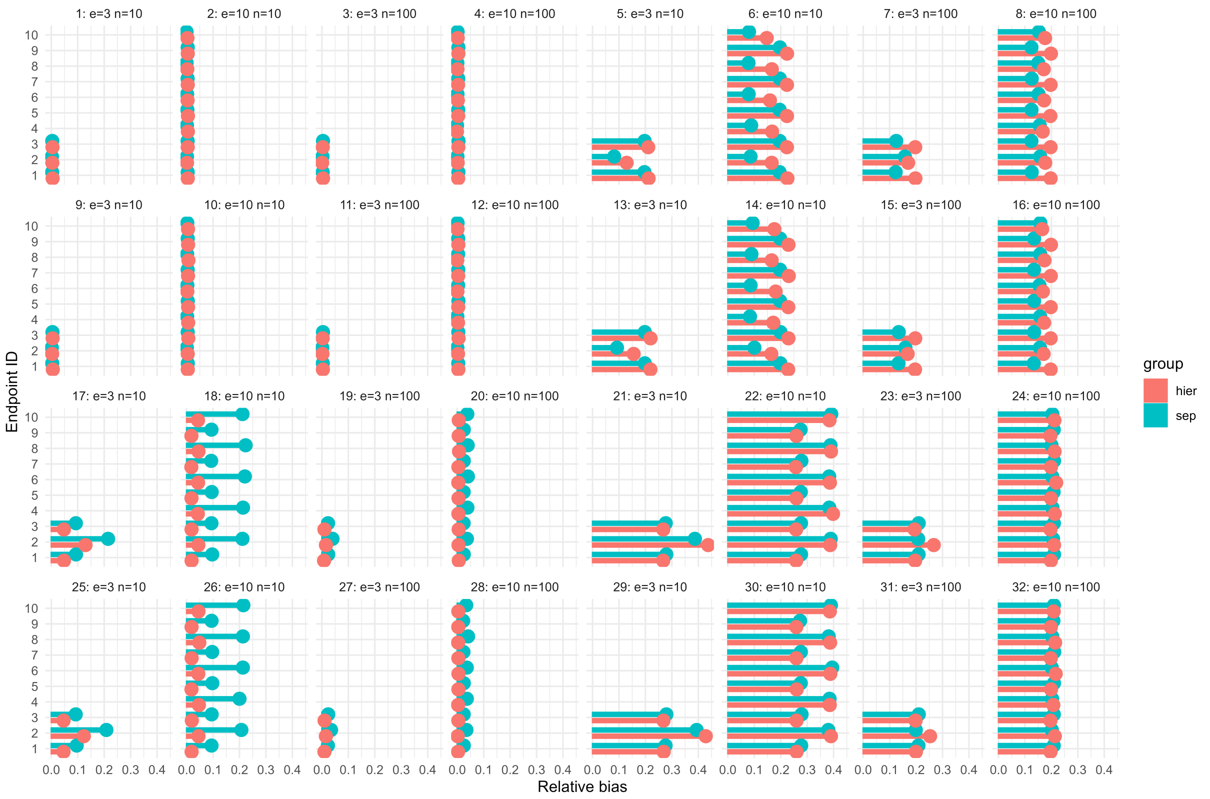

Figure 30 Lollipop plots of RMST relative bias estimates for the different simulated data scenarios. The separate model is coloured blue and the hierarchical model is coloured red.

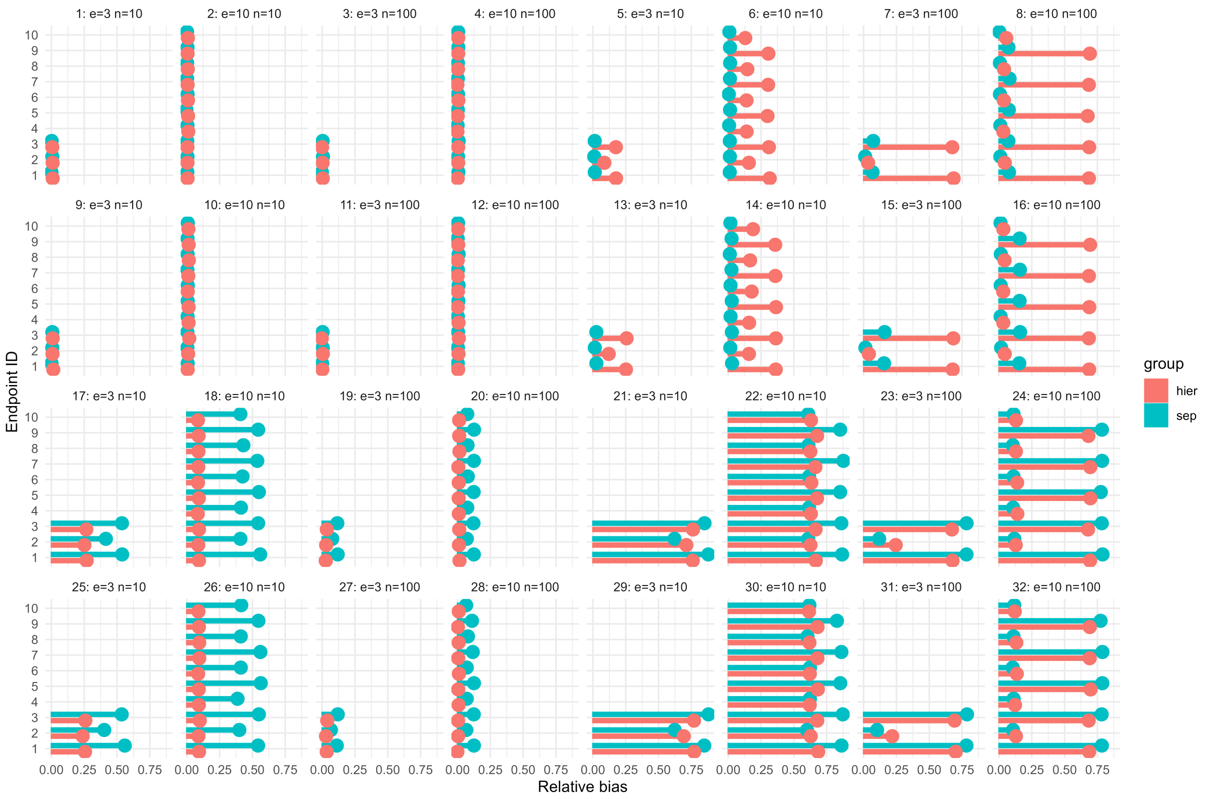

Figure 31 Lollipop plots of cure fraction relative bias estimates for the different simulated data scenarios. The separate model is coloured blue and the hierarchical model is coloured red.

10.1 | Zip plots of coverage

We present the zip plots for coverage of different target statistics. For each target statistic, we give the zip plots for the two specifications of latent survival curves as described in the main text, namely the Weibull with either rate $\lambda = 4$ or $\lambda = 1$. The plots are centred at the global mean value. The hierarchical model plots for cure fraction and RMST respectively are in Figure 34, 35, and Figure 38, 39. The separate model plots for cure fraction and RMST respectively are in Figure 32, 33, and Figure 36, 37.

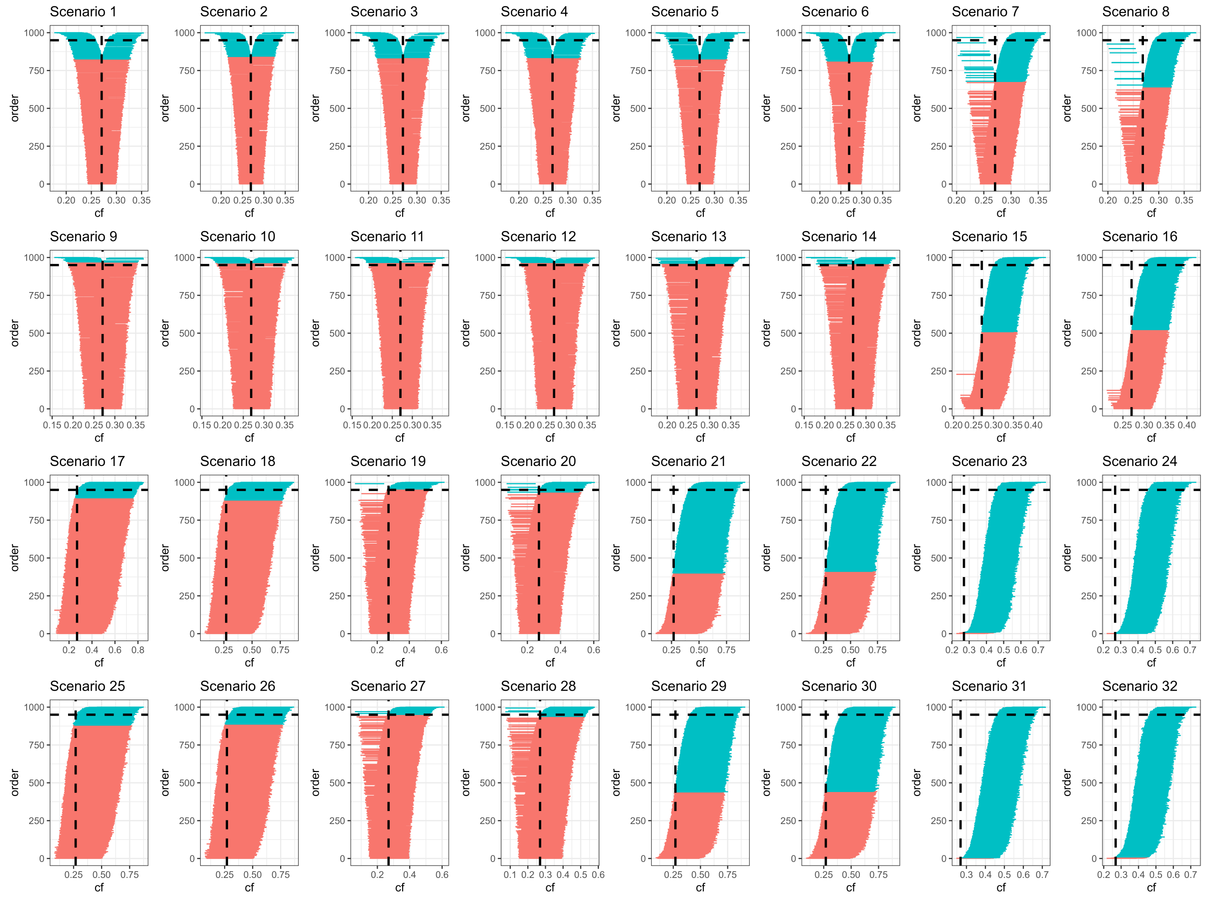

Figure 32 Zip plots of cure fraction over all separate model scenarios for latent Weibull survival curve with rate $\lambda = 4$. The horizontal dashed line is at the 95%.

□

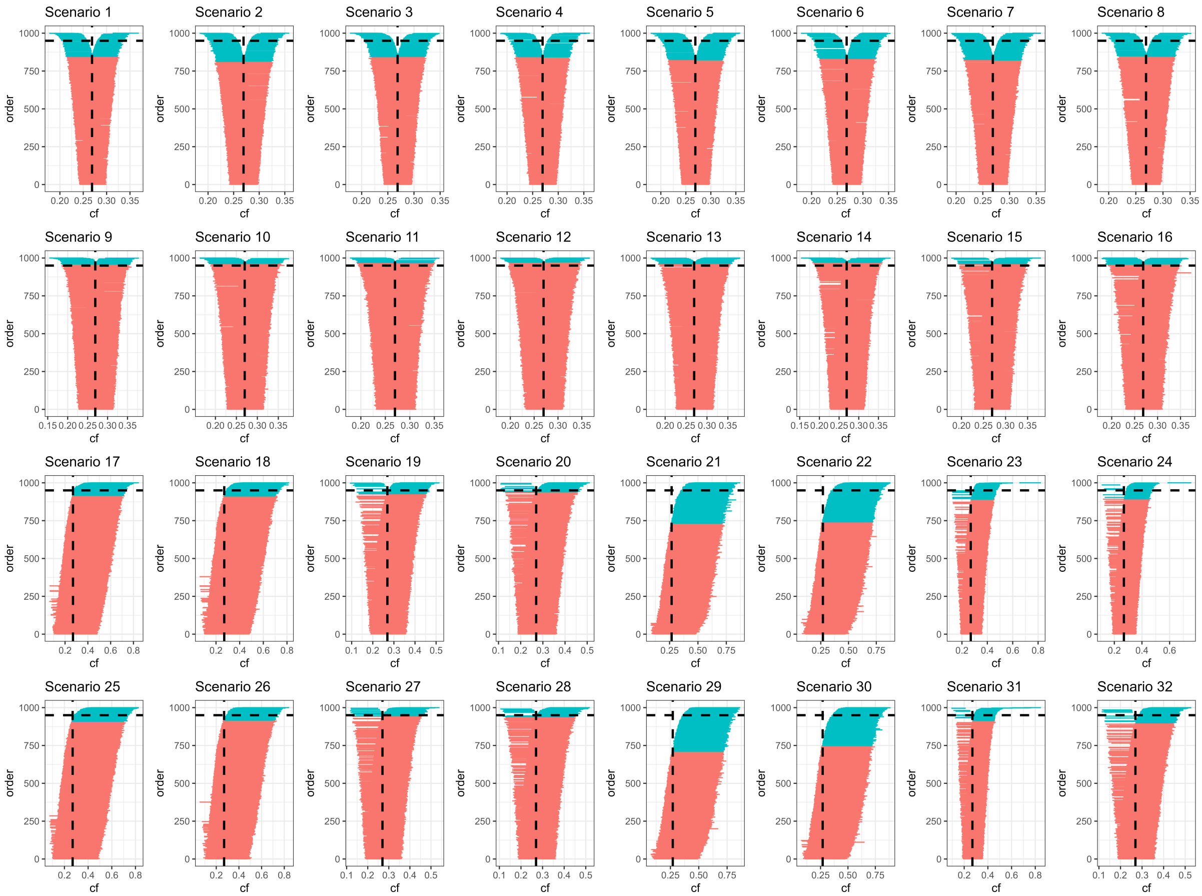

Figure 33 Zip plots of cure fraction over all separate model scenarios for latent Weibull survival curve with rate $\lambda = 1$. The horizontal dashed line is at the 95%.

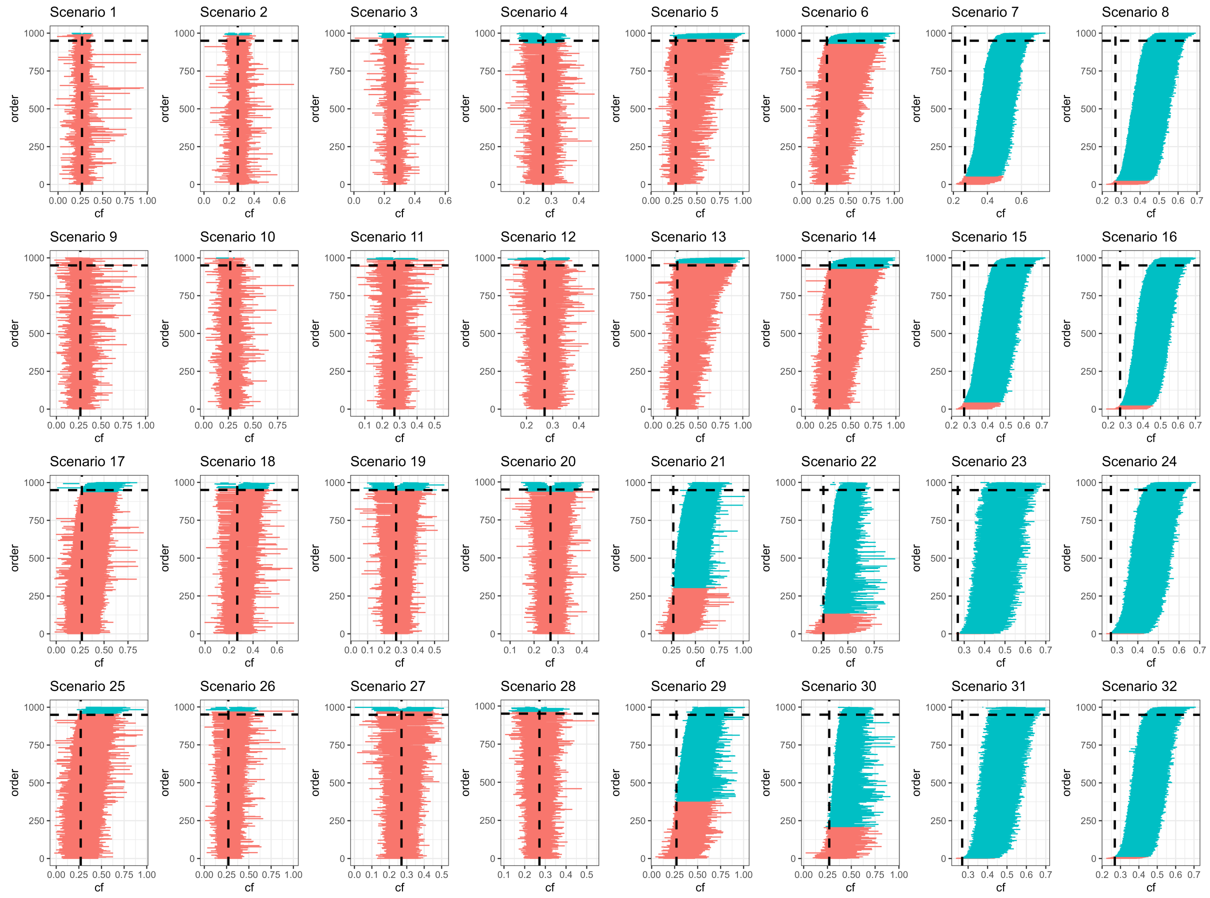

Figure 34 Zip plots of cure fraction over all hierarchical model scenarios for latent Weibull survival curve with rate $\lambda = 4$. The horizontal dashed line is at the 95%.

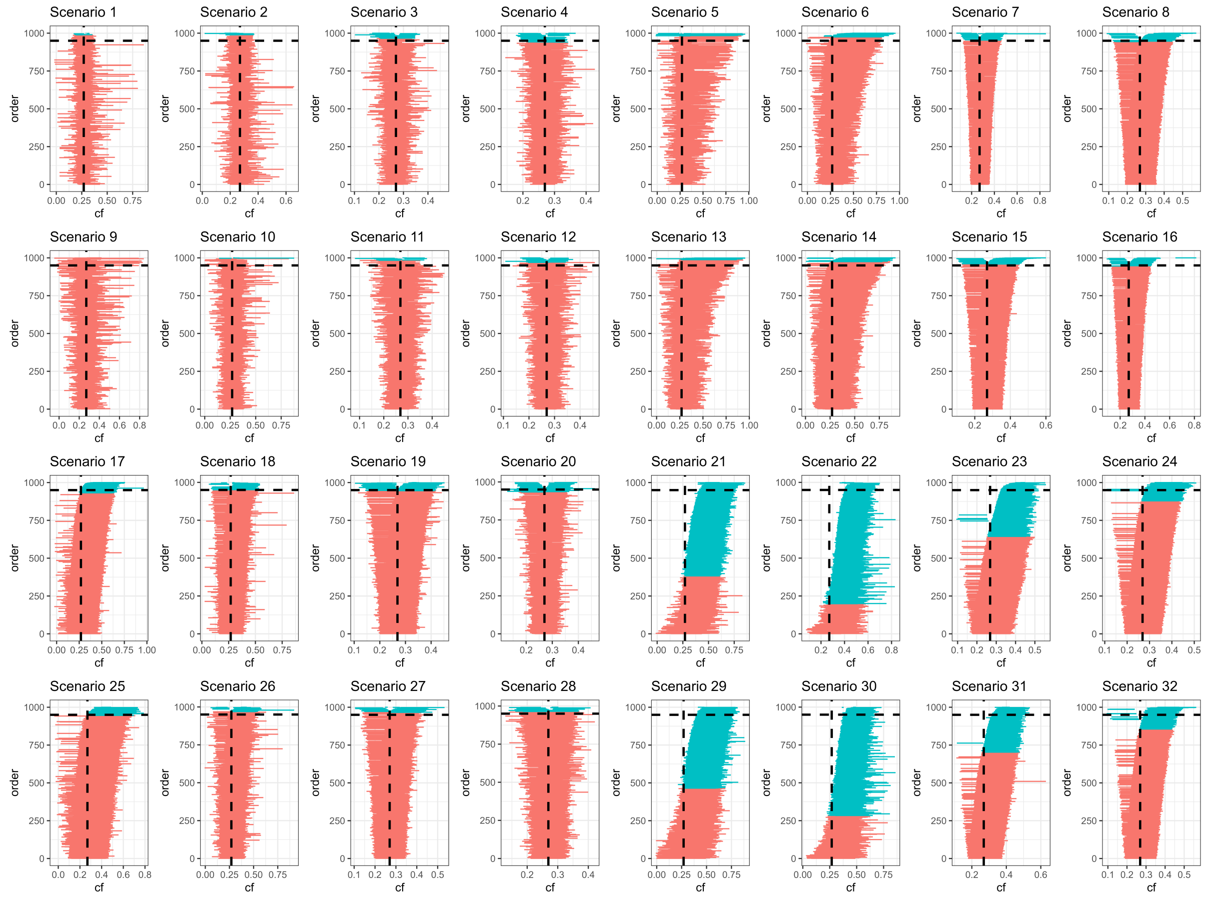

Figure 35 Zip plots of cure fraction over all hierarchical model scenarios for latent Weibull survival curve with rate $\lambda = 1$. The horizontal dashed line is at the 95%.

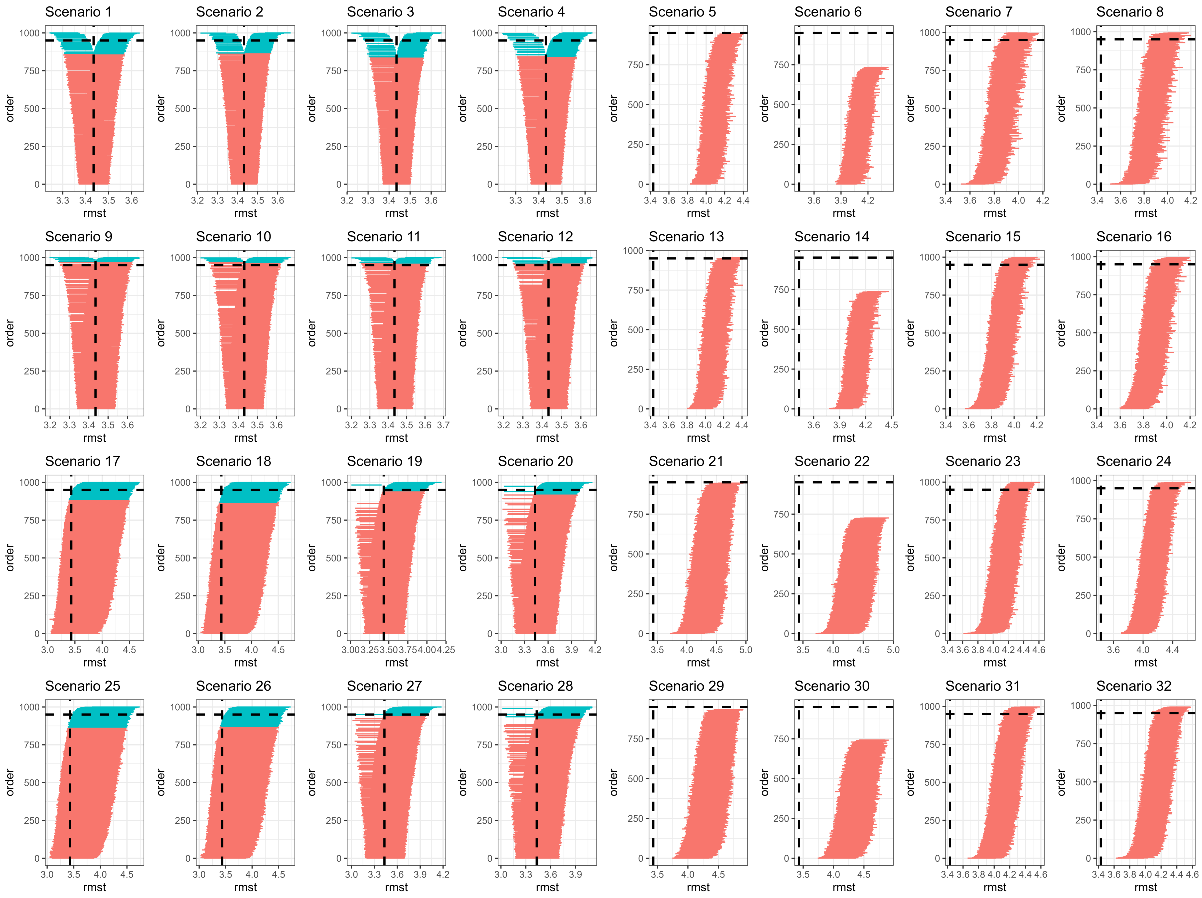

Figure 36 Zip plots of RMST over all separate model scenarios for latent Weibull survival curve with rate $\lambda = 4$. The horizontal dashed line is at the 95%.

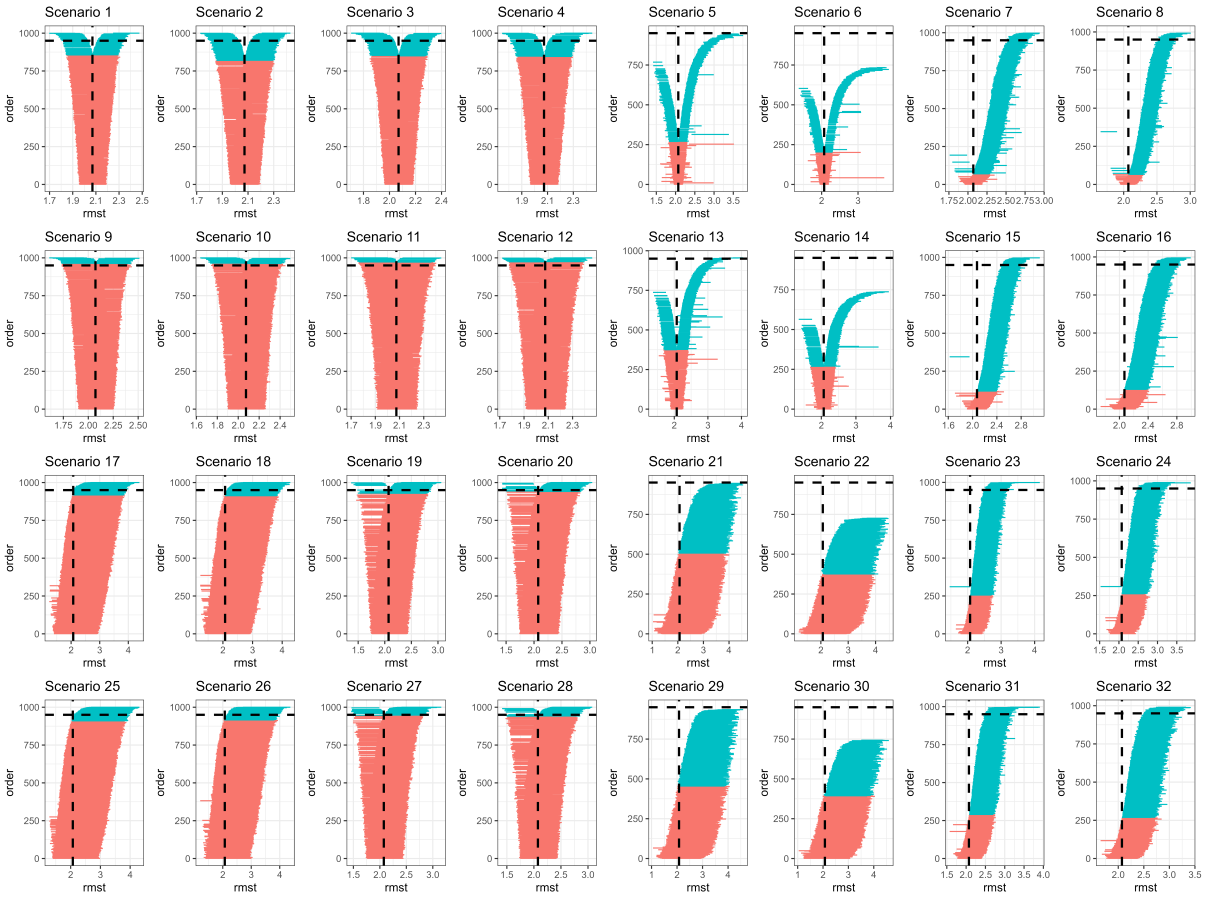

Figure 37 Zip plots of RMST over all separate model scenarios for latent Weibull survival curve with rate $\lambda = 1$. The horizontal dashed line is at the 95%.

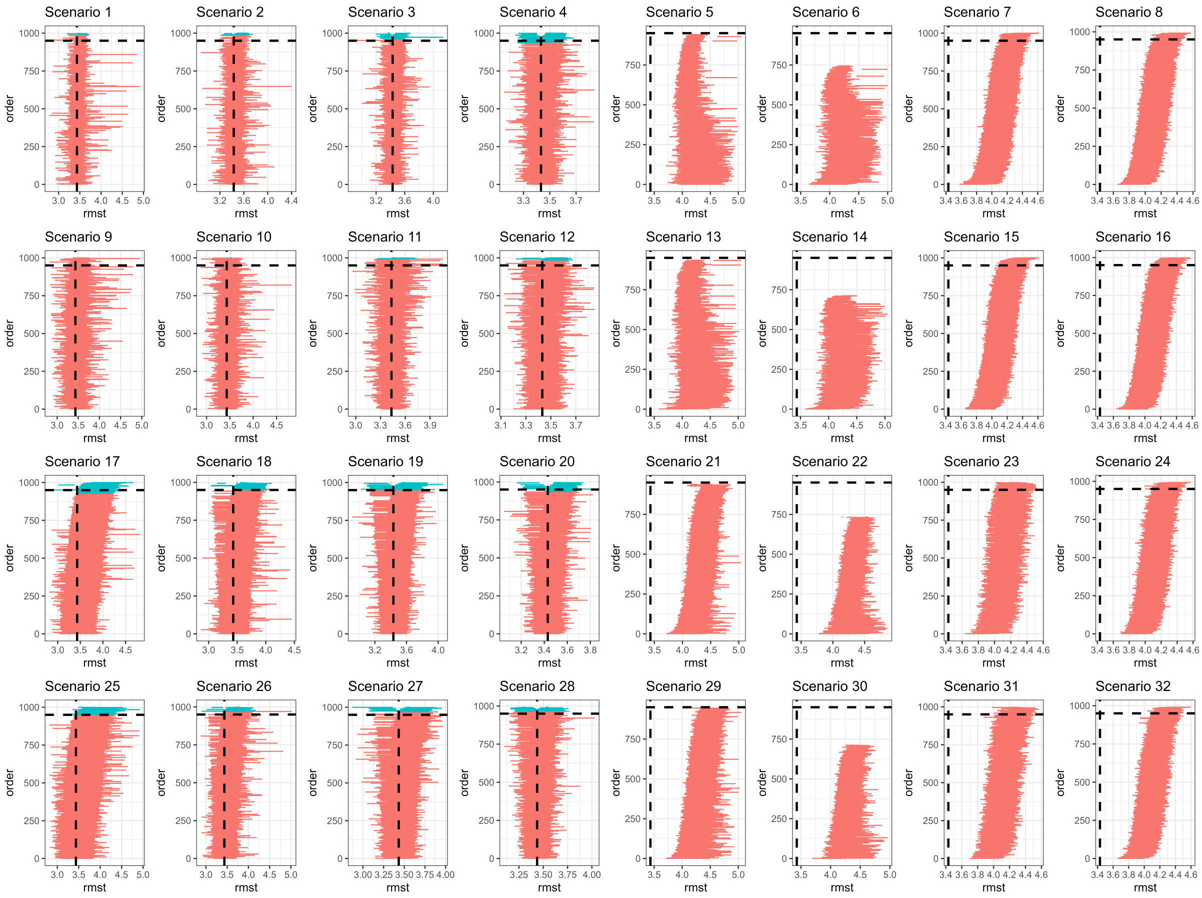

Figure 38 Zip plots of RMST over all hierarchical model scenarios for latent Weibull survival curve with rate $\lambda = 4$. The horizontal dashed line is at the 95%.

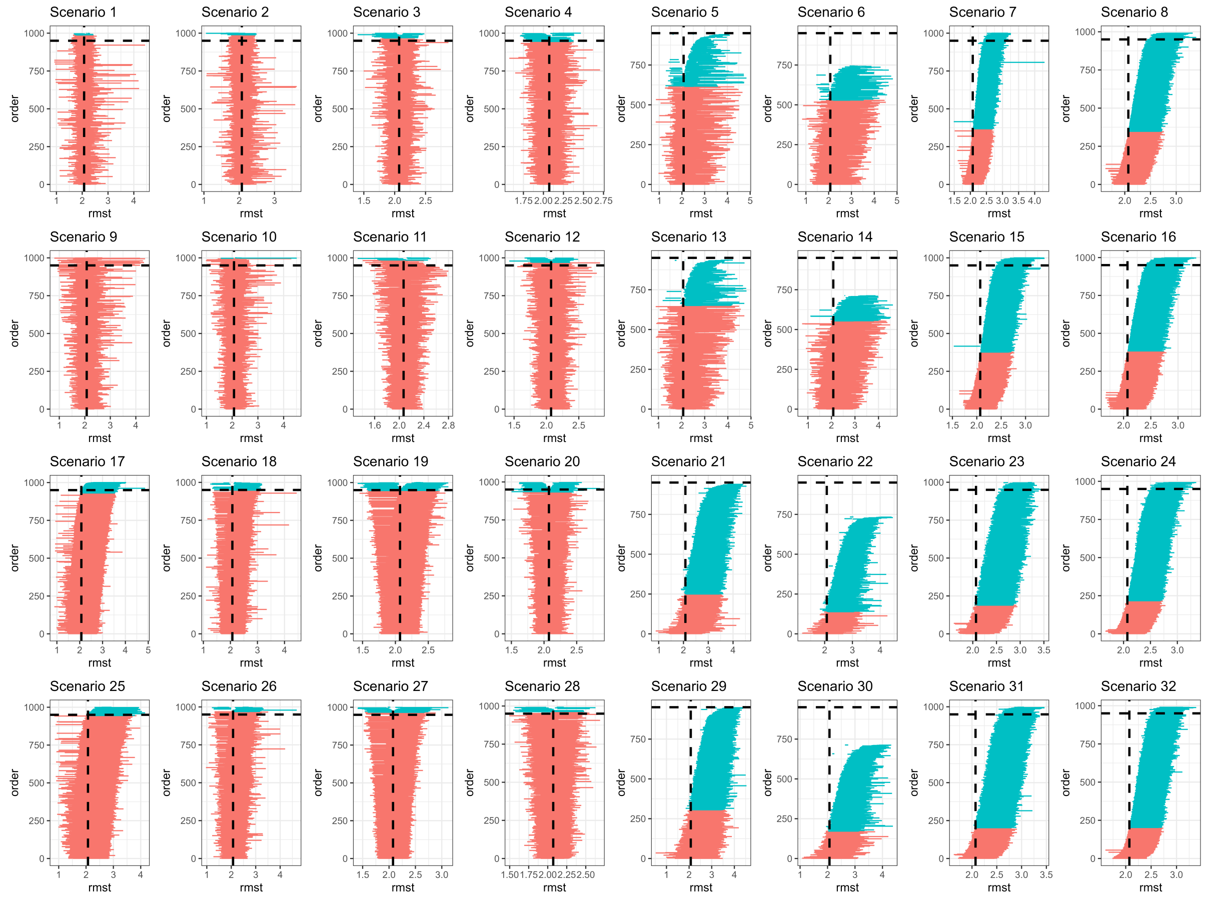

Figure 39 Zip plots of RMST over all hierarchical model scenarios for latent Weibull survival curve with rate $\lambda = 1$. The horizontal dashed line is at the 95%.
